# Environmental DNA metabarcoding primers for freshwater fish detection and quantification: In silico and in tanks

**DOI:** 10.1002/ece3.7658

**Published:** 2021-05-16

**Authors:** Lu Shu, Arne Ludwig, Zuogang Peng

**Affiliations:** ^1^ Key Laboratory of Freshwater Fish Reproduction and Development (Ministry of Education) School of Life Sciences Southwest University Chongqing China; ^2^ Department of Evolutionary Genetics Leibniz‐Institute for Zoo and Wildlife Research Berlin Germany; ^3^ Albrecht Daniel Thaer‐Institute Faculty of Life Sciences Humboldt University Berlin Berlin Germany

**Keywords:** environmental DNA, in silico, metabarcoding, primer selection, tank

## Abstract

Environmental DNA (eDNA) techniques refer to utilizing the organisms’ DNA extracted from environment samples to genetically identify target species without capturing actual organisms. eDNA metabarcoding via high‐throughput sequencing can simultaneously detect multiple fish species from a single water sample, which is a powerful tool for the qualitative detection and quantitative estimates of multiple fish species. However, sequence counts obtained from eDNA metabarcoding may be influenced by many factors, of which primer bias is one of the foremost causes of methodological error. The performance of 18 primer pairs for *COI*, *cytb*, *12S rRNA*, and *16S rRNA* mitochondrial genes, which are all frequently used in fish eDNA metabarcoding, were evaluated in the current study. The ribosomal gene markers performed better than the protein‐coding gene markers during in silico screening, resulting in higher taxonomic coverage and appropriate barcode lengths. Four primer pairs—AcMDB07, MiFish‐U, Ve16S1, and Ve16S3—designed for various regions of the *12S* and *16S rRNA* genes were screened for tank metabarcoding in a case study targeting six freshwater fish species. The four primer pairs were able to accurately detect all six species in different tanks, while only MiFish‐U, Ve16S1, and Ve16S3 revealed a significant positive relationship between species biomass and read count for the pooled tank data. The positive relationship could not be found in all species within the tanks. Additionally, primer efficiency differed depending on the species while primer preferential species varied in different fish assemblages. This case study supports the potential for eDNA metabarcoding to assess species diversity in natural ecosystems and provides an alternative strategy to evaluate the performance of candidate primers before application of eDNA metabarcoding in natural ecosystems.

## Graphical abstract

The maintenance of fish diversity is critical for aquatic ecosystem healthy, but in order to properly evaluate this, we must ensure that the tools used are properly evaluated and that we know how to identify problems or biases. As aquatic ecosystem management is a global issue, we feel that this research will have a broad audience in the world of freshwater and marine ecosystem research.

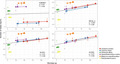

## 1 INTRODUCTION

Freshwater ecosystems make critical contributions to both human economies and societies, but their function and species diversity have been highly degraded by anthropogenic activities (Collen et al., 2013; Geist, 2011). Freshwater fish are vital to freshwater biodiversity and of substantial value to both water management and the public. Thus, efficient biological monitoring must be conducted to accurately assess changes in freshwater fish diversity and inform sustainable conservation policies and actions (Reid et al., 2019; Strayer & Dudgeon, 2010). Traditional fish monitoring methods, such as electrofishing and utilizing traps or nets, involve catching individuals for morphological characterization and visual counting in aquatic ecosystems (Bonar et al., 2009). These practices are time‐consuming, labor‐intensive, and destructive to ecological niches, as well as having low sensitivity and detection biases (Bayley & Peterson, 2001). Alternatively, environmental DNA (eDNA) techniques are noninvasive and increase sensitivity for rare fish detection compared with traditional methods (Evans, Li, et al., 2017; Evans, Shirey, et al., 2017; Pont et al., 2018; Shaw et al., 2016), although the cost efficiency of this technique is somewhat debatable (Smart et al., 2016). For these reasons, scientists have focused on the use of eDNA for monitoring threatened or invasive species and assessing fish biodiversity in the last decade (Coble et al., 2019; Harrison et al., 2019; Shu et al., 2020).

Environmental DNA techniques, which are based on sequence analysis of organisms’ DNA directly from environmental samples such as water, sediment, soil, or ice, can genetically identify target species without capturing actual organisms (Taberlet et al., 2012, 2018). Early eDNA detection for fish mainly relied on species‐specific qPCR assays to determine the presence or absence of a single species. However, more recent studies have used eDNA metabarcoding via high‐throughput sequencing (HTS) to simultaneously detect multiple fish species from a single water sample, improving the species detection efficiency of eDNA analysis (Coble et al., 2019; Shu et al., 2020). Furthermore, some studies found that eDNA sequence reads obtained from eDNA metabarcoding were positively associated with biomasses of target fish, in either the aquarium (Evans et al., 2015) or natural ecosystems (Hanfling et al., 2016; Thomsen et al., 2016; Ushio et al., 2018). These studies indicated that eDNA metabarcoding is a promising method for both the assessment of species diversity and biomass estimations of multiple fish species in fish communities.

However, the results of eDNA analyses can be affected by many different factors. Complex environmental factors, such as sunlight, water temperature, pH, microbial communities, and water flow can influence the ecology of eDNA (i.e., the origin, state, transport, and fate of DNA fragments in the environment), thus biasing detection (Hansen et al., 2018). Long‐time UV‐B exposure, high temperature, or high pH can increase eDNA decay rates, resulting in false‐negative detection of target species or hindering quantitative analysis (Barnes et al., 2014; Jo et al., 2019; Klobucar et al., 2017; Lacoursiere‐Roussel et al., 2016; Lance et al., 2017). Water flow has been shown to influence eDNA transport in lotic systems, resulting in eDNA of target species being transported meters to kilometers away and yielding false‐positive detection downstream (Deiner & Altermatt, 2014; Jane et al., 2015). Furthermore, technical factors, including biases in primer selection, sequencing, and sampling protocols, are considered the foremost methodological causes of eDNA detection or quantification error (Coble et al., 2019; Evans et al., 2015; Pawlowski et al., 2018; Thomsen & Willerslev, 2015). With respect to researcher‐controlled factors, careful primer selection is key to improving the efficiency and reliability of eDNA metabarcoding surveys.

Mitochondrial genes are standard markers for metabarcoding because of their taxonomic discriminatory power. Previous eDNA metabarcoding studies of fish both in freshwater and marine environments have targeted mitochondrial cytochrome B (*cytb*), cytochrome oxidase subunit I (*COI*), *12S rRNA*, and *16S rRNA* genes (Shu et al., 2020). *12S rRNA* markers are most commonly used in fish eDNA metabarcoding analysis followed by *16S rRNA* markers, including 12S‐V5 (*ca*. 106 bp) (Kelly et al., 2014; Riaz et al., 2011), MiFish‐U (*ca*. 170 bp) (Miya et al., 2015), and Teleo (*ca*. 65 bp) (Valentini et al., 2016) primer pairs designed for various regions of the *12S rRNA* sequence. As ribosomal genes have more universally conserved regions, their amplification enables detection of a larger proportion of target species and offers higher primer specificity, compared with protein‐coding genes (Bylemans et al., 2018; Collins et al., 2019; Deagle et al., 2014). However, shorter ribosomal markers have insufficient taxonomic resolution and are susceptible to detection biases (Hanfling et al., 2016; Thomsen et al., 2016). For example, the 12S‐V5 primer pair could not distinguish certain closely related species within the families Cyprinidae and Percidae (Hanfling et al., 2016). Moreover, each primer pair likely showed biased amplification for certain species because of species‐specific primer‐template mismatches (Elbrecht & Leese, 2015). Bylemans et al. (2018) found that the Teleo primer pair had reduced amplification efficiencies for the common carp and did not reflect actual species relative abundance from carp sequence reads. To reduce the bias resulting from single genetic markers, a number of studies have employed multiple genetic markers to increase the probability of species detection and accuracy of species quantification (Evans, Li, et al., 2017; Evans, Shirey, et al., 2017; Evans et al., 2015; Hanfling et al., 2016; Li, Hatton‐Ellis, et al., 2018; Li, Evans, et al., ; Olds et al., 2016; Shaw et al., 2016).

The performance of eDNA metabarcoding primers varies when detecting species biodiversity in different geographic regions; thus, it is difficult to propose an optimal genetic marker or the most appropriate suite of primers for universal use. For this reason, primer testing is necessary before beginning field surveys. The efficacy of different eDNA metabarcoding primers targeting fish species have been compared in tank experiments (Evans et al., 2015), and in both in silico and in vitro studies (Bylemans et al., 2018; Collins et al., 2019; Zhang et al., 2020). These studies proposed preferentially choosing primers for future eDNA applications and workflows for assessing metabarcoding primers in natural ecosystems. However, the studies either included only a small number of existing primers for fish, or the complex field environments made it difficult to determine whether the primers or other factors caused detection and quantification biases. Therefore, a clear evaluation of all available metabarcoding primers targeting fish when screened in silico and used under controlled tank conditions is required.

In the current study, four controlled tanks consisting of six freshwater fish species were used as a case study to identify optimal primers for metabarcoding analyses. Eighteen universal primer pairs for fish eDNA metabarcoding from the mitochondrial genes *COI*, *cytb*, *12S rRNA,* and *16S rRNA* were thus evaluated both in silico and under controlled tank conditions, providing important information for future primer selection prior to eDNA metabarcoding surveys in the field.

## 2 METHODS

### 2.1 In silico *screening of metabarcoding primers*

All the available eDNA metabarcoding primers targeting fish species were summarized in a previous review (Shu et al., 2020). The 18 primer pairs for mitochondrial genetic markers are shown in Table 1. PrimerMiner (Elbrecht & Leese, 2016) and PrimerTree (Cannon et al., 2016) packages in R v3.5.3 (www.r‐project.org) were used to estimate taxonomic coverage, resolution, and amplification success of the metabarcoding primers, as described previously by Bylemans et al. (2018). Taxonomic coverage analysis evaluated the universality of the metabarcoding primers amplifying the target taxonomic group, while taxonomic resolution analysis aimed to evaluate the discriminatory ability of the metabarcoding primers for differentiating among taxa. Amplification success analysis was used to evaluate primer specificity and the effect of primer bias on amplification. Higher taxonomic coverage and appropriate barcode lengths, higher taxonomic resolution, and specific and unbiased amplification were identified as the selection criteria for well‐performing primers in silico.

**TABLE 1:** Primer pairs used in silico evaluation^a^

Target gene	Name in literature	Name in this study	Direction	Primers sequence 5’−3’
*COI*	PS1‐F/R	PS1	Forward	ACCTGCCTGCCGTATTTGGYGCYTGRGCCGGRATAGT
			Reverse	ACGCCACCGAGCCARAARCTYATRTTRTTYATTCG
*Cytb*	FishCBL/FishCBR	FishCB1	Forward	TCCTTTTGAGGCGCTACAGT
			Reverse	GGAATGCGAAGAATCGTGTT
	Fish2CBL/Fish2bCBR	FishCB2	Forward	ACAACTTCACCCCTGCAAAC
			Reverse	GATGGCGTAGGCAAACAAGA
	L14841/H15149	VeCB1	Forward	AAAAAGCTTCCATCCAACATCTCAGCATGATGAAA
			Reverse	AAACTGCAGCCCCTCAGAATGATATTTGTCCTCA
	L14912/H15149c	VeCB2	Forward	AAAAACCACCGTTGTTATTCAACTA
			Reverse	GCCCCTCAGAATGATATTTGTCCTCA
	L14912/H15149	VeCB3	Forward	TTCCTAGCCATACAYTAYAC
			Reverse	GGTGGCKCCTCAGAAGGACATTTGKCCYCA
*12S rRNA*	12S‐V5	12S‐V5	Forward	ACTGGGATTAGATACCCC
			Reverse	TAGAACAGGCTCCTCTAG
	Ac12s	Ac12s	Forward	ACTGGGATTAGATACCCCACTATG
			Reverse	GAGAGTGACGGGCGGTGT
	Am12s	Am12s	Forward	AGCCACCGCGGTTATACG
			Reverse	CAAGTCCTTTGGGTTTTAAGC
	MiFish‐U	MiFish‐U^b^	Forward	GTCGGTAAAACTCGTGCCAGC
			Reverse	CATAGTGGGGTATCTAATCCCAGTTTG
	Teleo	Teleo	Forward	ACACCGCCCGTCACTCT
			Reverse	CTTCCGGTACACTTACCATG
	AcMDB07	AcMDB07^b^	Forward	GCCTATATACCGCCGTCG
			Reverse	GTACACTTACCATGTTACGACTT
*16S rRNA*	Ac16s	Ac16s	Forward	CCTTTTGCATCATGATTTAGC
			Reverse	CAGGTGGCTGCTTTTAGGC
	L2513/H2714	Ve16S1^b^	Forward	GCCTGTTTACCAAAAACATCAC
			Reverse	CTCCATAGGGTCTTCTCGTCTT
	Ve16s	Ve16S2	Forward	CGAGAAGACCCTATGGAGCTTA
			Reverse	AATCGTTGAACAAACGAACC
	Vert‐16S‐eDNA	Ve16S3^b^	Forward	AGACGAGAAGACCCYDTGGAGCTT
			Reverse	GATCCAACATCGAGGTCGTAA
	Fish‐specific primer set	Fish16S1	Forward	CGAGAAGACCCTWTGGAGCTTIAG
			Reverse	GGTCGCCCCAACCRAAG
	Fish16SF/D,16S2R‐degenerate	Fish16S2	Forward	GACCCTATGGAGCTTTAGAC
			Reverse	CGCTGTTATCCCTADRGTAACT

^a^ See the supplementary materials of Shu et al. (2020) for the complete list of primers amplicon length, annealing temperatures and references.

^b^ Four primer pairs were screened in silico for tank eDNA metabarcoding analyses.

First, the taxonomic coverage of each primer pair was calculated as the number of species recovered from known fish mitochondrial genome sequences using the MitoFish database (2,838 whole mitogenome sequences) (Iwasaki et al., 2013), using a maximum mismatch of 3 and gap cost of 0. Three mismatches were a reasonable value that minimized amplification bias for the reference database (Taberlet et al., 2018). Initial optimal primer pairs were selected when the amplified barcodes contained more than 2,000 fish species (recovered from the MitoFish database) and a mean length >150 bp and <350 bp. In principle, the number of species amplified by a primer should be as large as possible; however, the practical number of recovered species by primers for in silico analysis ranged from 3 to 2,675. Therefore, we chose 2,000 fish species as a threshold to determine primer pairs for further analysis. Moreover, amplicon length is a trade‐off between increasing the ability to recover highly degraded eDNA fragments in water samples and ensuring sufficient taxonomic resolutions. Second, the taxonomic resolution of the barcodes for the initial selected primer pairs was estimated as the average number of bp differences between species, per 100 bases, using the MitoFish database. Third, the amplification success of the initial selected primer pairs was evaluated using sequences from the MitoFish database based on positive correlations between primer‐template mismatches and amplification success, with threshold values ranging from 0 to 15,000. Theoretically, the more primer‐template mismatches (higher threshold values) allowed, the higher the level of amplification success. Finally, the initial selected primer pairs were used for subsequent tank tests to evaluate their performance for species detection and abundance or biomass estimations.

### 2.2 Tank eDNA metabarcoding

#### 2.2.1 Experimental design

Six fish species from freshwater aquatic ecosystems were obtained from local commercial markets and field ponds in Chongqing, China: *Carassius auratus*, *Cyprinus carpio*, *Gambusia affinis*, *Hypophthalmichthys molitrix*, *Misgurnus anguillicaudatus*, and *Pseudorasbora parva*. *C. carpio*, *H. molitrix,* *P. parva,* and *G. affinis* are invasive species in America, Europe, and Asia that have previously been the focus of eDNA surveys (Robinson et al., 2019; Wilson et al., 2014). Five experimental tanks and all equipment were decontaminated using a 10% bleach solution prior to the experiment. To avoid contamination with foreign fish DNA, fish were reared in single‐species tanks for 1 week and then transferred to four 100‐L tanks (Tanks 1–4), with four different treatments: high total density and even relative abundance; low total density and even relative abundance; high total density and skewed relative abundance; low total density and skewed relative abundance (Table 2). Even relative abundance in the high‐ and low‐density tanks included 6 and 3 individuals per species, respectively. Skewed relative abundance in the high‐ and low‐density tanks was determined by choosing two random sets of numbers using the “*runif”* function in R package, based on lognormal distribution observed in naturally occurring fish assemblages (Magurran & Henderson, 2003). The tanks were constructed with reference to methodology previously described by Evans et al. (2015). In addition, a negative control tank was filled with 100 L tap water but no fish. Fish were maintained under a 12 hr light/12 hr dark cycle and 21 ± 1°C water temperature and were subjected to fasting 1 week prior to day 0 until the end of the trial to avoid contamination by foreign fish DNA from feed and feces. Dead individuals were immediately removed from tanks and replaced with healthy fish of the same species from the single‐species tanks (Table S1). The wet weights of individual fish from each tank were measured (nearest 0.01 g) at the end of the experiment and the biomass in each tank was calculated (Table 2).

**TABLE 2:** Abundance and biomass of each species in four experimental tanks

Species	High density, even abundance		Low density, even abundance		High density, skewed abundance		Low density, skewed abundance	
	Tank 1 (100 L)		Tank 2 (100 L)		Tank 3 (100 L)		Tank 4 (100 L)	
Scientific name	Abundance	Biomass (g)	Abundance	Biomass (g)	Abundance	Biomass (g)	Abundance	Biomass (g)
*Carassius auratus*	6	94.35	3	47.93	4	59.26	8	118.58
*Cyprinus carpio*	6	86.54	3	31.55	8	93.31	2	29.03
*Gambusia affinis*	6	2.29	3	1.04	18	6.43	10	3.53
*Hypophthalmichthys molitrix*	6	55.79	3	29.52	2	24.76	3	30.39
*Misgurnus anguillicaudatus*	6	48.93	3	41.74	5	50.84	2	32.66
*Pseudorasbora parva*	6	6.06	3	4.68	10	10.13	3	5.36

#### 2.2.2 Sample collection and eDNA extraction

Three replicate 200‐ml samples of water were collected using sterilized bottles from each tank, including the negative control tank, both before and 5 days after transferring the fish to the experimental tanks. In total, 30 water samples, 18 of which were negative controls, were used for further processing. The negative controls were tested for evidence of contamination during the experimental procedures. Subsequently, water samples were filtered using 0.45‐µm mixed cellulose acetate and nitrate (MCE) filters (Whatman International Ltd.). eDNA was extracted from the filters using the PowerWater DNA Isolation Kit (MO BIO Laboratories Inc.) following the manufacturer’s protocol. All eDNA extracts were stored at −20°C until PCR amplification.

#### 2.2.3 PCR amplification and HTS sequencing

Two‐step PCR amplification was performed for Illumina paired‐end library preparation. The first PCR was performed in a total volume of 20 μl, including 10 ng of DNA template, 0.8 μl each of the forward and reverse primers (10 μM), 4 μl of 5 × FastPfu Buffer (TransGen Biotech Ltd.), 2 μl of 2.5 mM dNTPs, 0.4 μl of FastPfu Polymerase (both TransGen Biotech Ltd.; 2.5 units/μl), and dd H_2_O added to 20 μl. The thermal cycling profile was 95°C for 5 min; 27 cycles at 95°C for 30 s, annealing temperature (*T*_a_) for 30 s, and 72°C for 45 s; followed by 72°C for 10 min. The optimal *T*_a_ for each primer pair was determined using gradient PCR spanning a range from 55 to 65°C based on temperatures used in the original design papers. The best amplification visualized by agarose gel electrophoresis revealed the optimal *T*_a_ was 55, 58, 60, and 60°C for the AcMDB07, MiFish‐U, Ve16S1, and Ve16S3 primer pairs, respectively. For each sample, triplicate PCRs were performed and pooled after amplification to reduce potential PCR bias. The first PCR products were estimated using 2% agarose gels and then purified using the AxyPrep DNA Gel Extraction Kit (Axygen Biosciences). The purified first PCR products were used as templates for the second PCR. The negative controls were not analyzed further because no target DNA was amplified from the negative controls.

The second PCR was used to add Illumina adaptor and index sequences to both amplicon ends. The second PCR volume was 50 μl, including 5 μl of DNA template, 5 μl each of the forward and reverse primers (10 μM), 25 μl of 2 × KAPA HiFi HotStart ReadyMix (Roche Sequencing and Life Science), and 10 μl PCR‐grade water. The thermal cycling profile was 95°C for 3 min; eight cycles of 95°C for 30 s, 55°C for 30 s, and 72°C for 30 s; followed by 72°C for 5 min. The indexed second PCR products were pooled in equal volumes and purified using the AxyPrep DNA Gel Extraction Kit. The DNA concentrations of the libraries were checked using a Qubit dsDNA HS Assay Kit (Thermo Fisher Scientific) and a Qubit Fluorometer (Thermo Fisher Scientific). The libraries, based on the different lengths of the amplicons, were sequenced on a HiSeq 2500 system (Illumina Inc.) for paired‐end 2 × 250 bp reads or a NovaSeq 6000 system for paired‐end 2 × 150 bp reads (Illumina Inc). To reduce the bias effects of sequencing depth on each sample, the expected number of total reads per sample was set to 500,000.

#### 2.2.4 Bioinformatic analyses

Using Trimmomatic v.0.36 (Bolger et al., 2014) and Flash programs (Magoc & Salzberg, 2011), the paired‐end reads were trimmed and merged with Phred quality scores ≥20 and minimum overlapping of 10. Erroneous merged reads containing ambiguous bases or those shorter than 100 bp were filtered out. A maximum mismatch of 2 was allowed between primer and template. Sequencing adaptors were removed from each read using the TagCleaner tool. Sequences were clustered into operational taxonomic units (OTUs) at 97% identity using Usearch v.10 (Edgar et al., 2011). OTUs with a total read count <10 in the whole data were filtered out. Finally, we assigned taxonomic groups to OTUs with ≥97% identity and ≤10^−5^ E‐value using the Blastn tool (Camacho et al., 2009) and the custom‐made reference database built for this study. Reference sequences containing one complete mitochondrial genome sequence for each species were obtained from GenBank: *C*. *auratus* (KJ874430), *C. carpio* (KU159761), *G. affinis* (AP004422), *H. molitrix* (EU315941), *M. anguillicaudatus* (DQ026434), and *P. parva* (JF802126).

Read numbers of each eDNA sample were standardized to ensure comparable numbers of reads per species among samples and primer pairs, since the total number of DNA reads varied among samples and sequencing runs. All eDNA samples were resampled to randomly select 833,329 reads per sample using QIIME v.1.9.0 (Caporaso et al., 2010), which was the smallest total number of reads found in one sample. All taxa detected from the initial dataset were still found after resampling, and the proportions of reads per species in individual samples were identical before and after resampling. Standardized reads for each species detected with the four primer pairs in individual samples were used for the correlation analyses between abundance or biomass of the fish species and read counts, and the variation analyses of species‐level sequence abundance among the four primer pairs. The average number of standardized reads for each species detected from the three replicate water samples per tank using each primer pair was used for plotting the bar chart and heatmap, which illustrate species compositions and relative sequence abundances detected using the four primer pairs in each tank. Species accumulation curves were plotted for each primer pair with sequencing depth using the “*vegan”* function in R, to determine sequencing sensitivity.

#### 2.2.5 Statistical analyses

Bar histograms were plotted using the “*barplot”* function in R, according to the average number of standardized reads and the abundance and biomass of each species detected with each primer pair in each tank. To evaluate the performance of the multispecies eDNA quantification for the four primer pairs, the linear mixed‐effects (LME) model in the form of linear regression was used to identify correlations between abundance or biomass of the fish species and read count obtained by each primer pair. LME model estimations were applied using the *“lmer”* function in the R package *lmerTest*. Considering the different potential sources of variation for our data (i.e., species reads detected using the four primer pairs were nested within samples that were nested within tanks), different datasets were used in correlation analyses. Pooled data from all four tanks (6 species × 4 tanks × 3 replicates = 72 data points per primer pair) were used to estimate the correlations between abundances or biomass and read counts of all species combined within the four tanks for each primer pair, with read counts as response variables, abundances or biomass as fixed effects, and species as random effects. Species‐specific data (4 tanks × 3 replicates = 12 data points per primer pair for each species) were used to estimate the correlations between biomass and read counts of each species within the four tanks for each primer pair, with read counts as response variables, biomass as fixed effects, and tanks as random effects. The data from each separate tank (6 species × 3 replicates = 18 data points per primer pair at each tank) were used to estimate the correlations between biomass and read counts of all species combined for each tank detected using each primer pair, with read counts as response variables, biomass as fixed effects, and species as random effects. The data were not normally distributed when evaluating residual‐expected value plots for model fit. Therefore, eDNA read counts were log10‐transformed to improve model fit and appropriately describe the observed relationship.

The relative sequence abundance of each fish obtained using the four primer pairs in each tank was visualized in a heatmap using the *pheatmap* package in R based on the average number of standardized reads in three replicate samples. White’s *t* test (White et al., 2009) was used to assess species‐level sequence abundance differences between the four primer pairs using STAMP software v2.1.3 (Parks et al., 2014), based on replicate read counts. Boxplots were plotted only when significant differences (*p* < .05) in species reads were found between two primer pairs.

## 3 RESULTS

### 3.1 In silico *primer evaluation*

**FIGURE 1:**
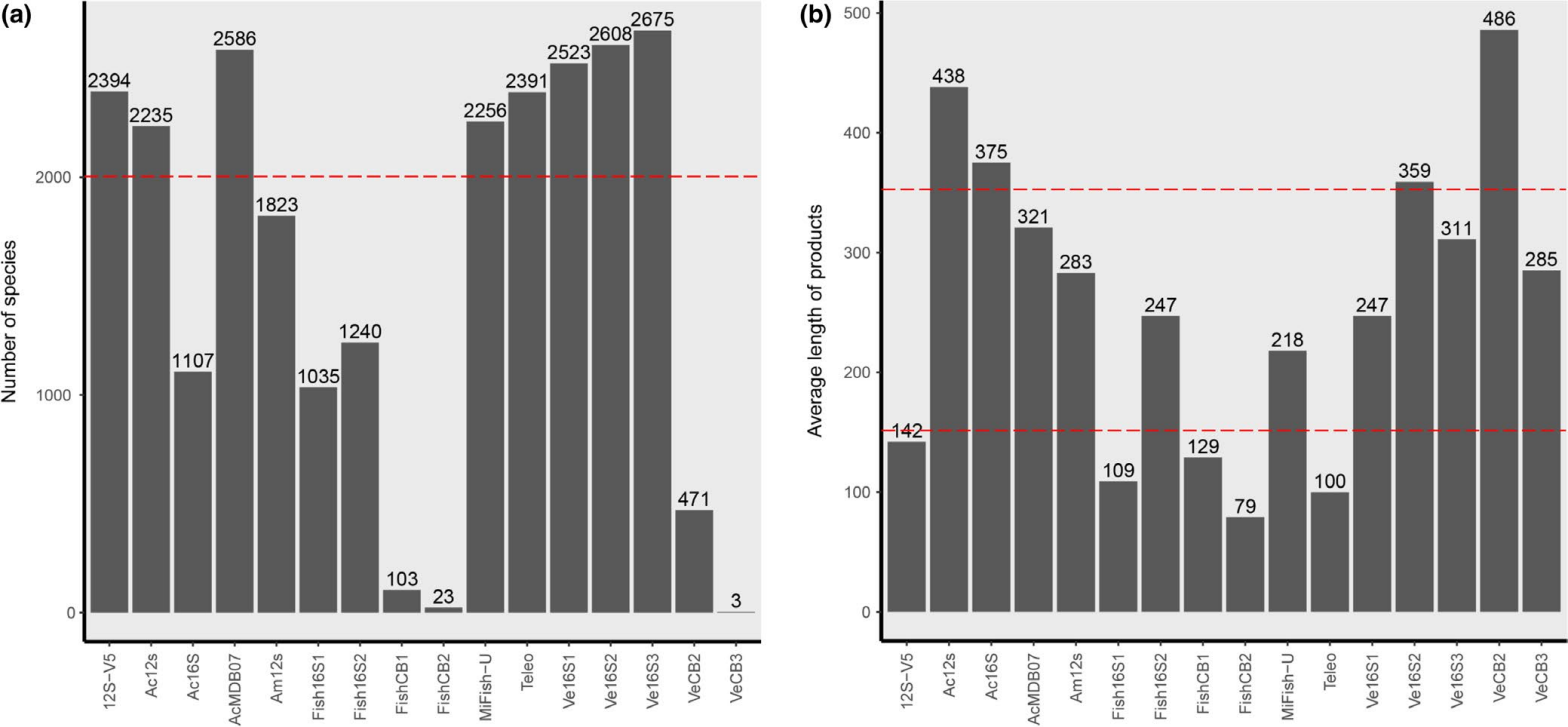
Initial screening results of the primer pairs using the R package *PrimerMiner*. The dashed red lines represent the threshold values for each statistic. (a) The taxonomic coverage for each primer pair was estimated as the number of fish recovered using the MitoFish database. (b) The average barcode length of each primer pair was calculated using the amplified sequence lengths in silico

The taxonomic coverage and average barcode lengths of the 18 eDNA metabarcoding primer pairs (Table 1) used for in silico analyses are shown in Figure 1. Sixteen of the primer pairs recovered fish species sequences from the MitoFish database (Figure 1a), and eight primer pairs exceeded the threshold values set for taxonomic coverage. Among them, four primer pairs had amplifying barcode lengths within the threshold range and were considered suitable for further analyses (Figure 1b). The four selected primer pairs were designed to amplify fragments of *12S rRNA* (AcMDB07 and MiFish‐U) and *16S rRNA* (Ve16S1 and Ve16S3) mitochondrial genes (Bylemans et al., 2018; Kitano et al., 2007; Miya et al., 2015; Vences et al., 2016), with average amplification lengths (containing primers) of 321, 218, 247, and 311 bp, respectively. The statistics obtained from the taxonomic resolution and primer specificity analyses for the four primer pairs are shown in Figure [Link], [Link]. Among the four primer pairs, AcMDB07 had the highest taxonomic resolution of the amplification barcodes (36 bp differences between amplified barcodes per 100 bases), followed by Ve16S3 (32 bp differences between amplified barcodes per 100 bases), and MiFish‐U (8 bp differences between amplified barcodes per 100 bases), while Ve16S1 had the lowest taxonomic resolution (1 bp differences between amplified barcodes per 100 bases) (Figure [Link], [Link]a). The amplification success analyses did not show differences in the amplification of fish mitochondrial gene sequences among the four primer pairs using the MitoFish database (Figure [Link], [Link]b).

### 3.2 Metabarcoding analyses

Species accumulation curves showing species diversity (OTU richness) were constructed, with increasing numbers of sequence reads tending to reach a plateau, which indicated that sequencing depths were sufficient to recover the amplified species for the four primer pairs (Figure S2). Illumina HTS of the 48 libraries (3 sampling replicates from 4 tanks for 4 primer pairs) yielded 192.62 million raw reads, including 27.50, 69.69, 59.49, and 35.93 million reads for AcMDB07, MiFish‐U, Ve16S1, and Ve16S3, respectively. After quality filtering, 12.44, 31.05, 27.62, and 16.63 million clean reads were acquired for AcMDB07, MiFish‐U, Ve16S1, and Ve16S3, respectively. Then, each eDNA sample was standardized to 833,329 reads, which was the smallest total number of reads found in one sample of the replicates. After BLAST searches for taxonomic assignments, 8,204,762 (82.05%), 9,102,232 (91.02%), 6,630,368 (66.30%), and 8,903,288 (89.03%) sequences from the 12 replicate samples were assigned to the known fish species using custom‐made references for the AcMDB07, MiFish‐U, Ve16S1, and Ve16S3 primer pairs, respectively (Table [Link], [Link]). The average reads for each species of each triplicate sample detected with four primer pairs in four tanks were shown in Table [Link], [Link].

### 3.3 Species detection and quantification

**FIGURE 2:**
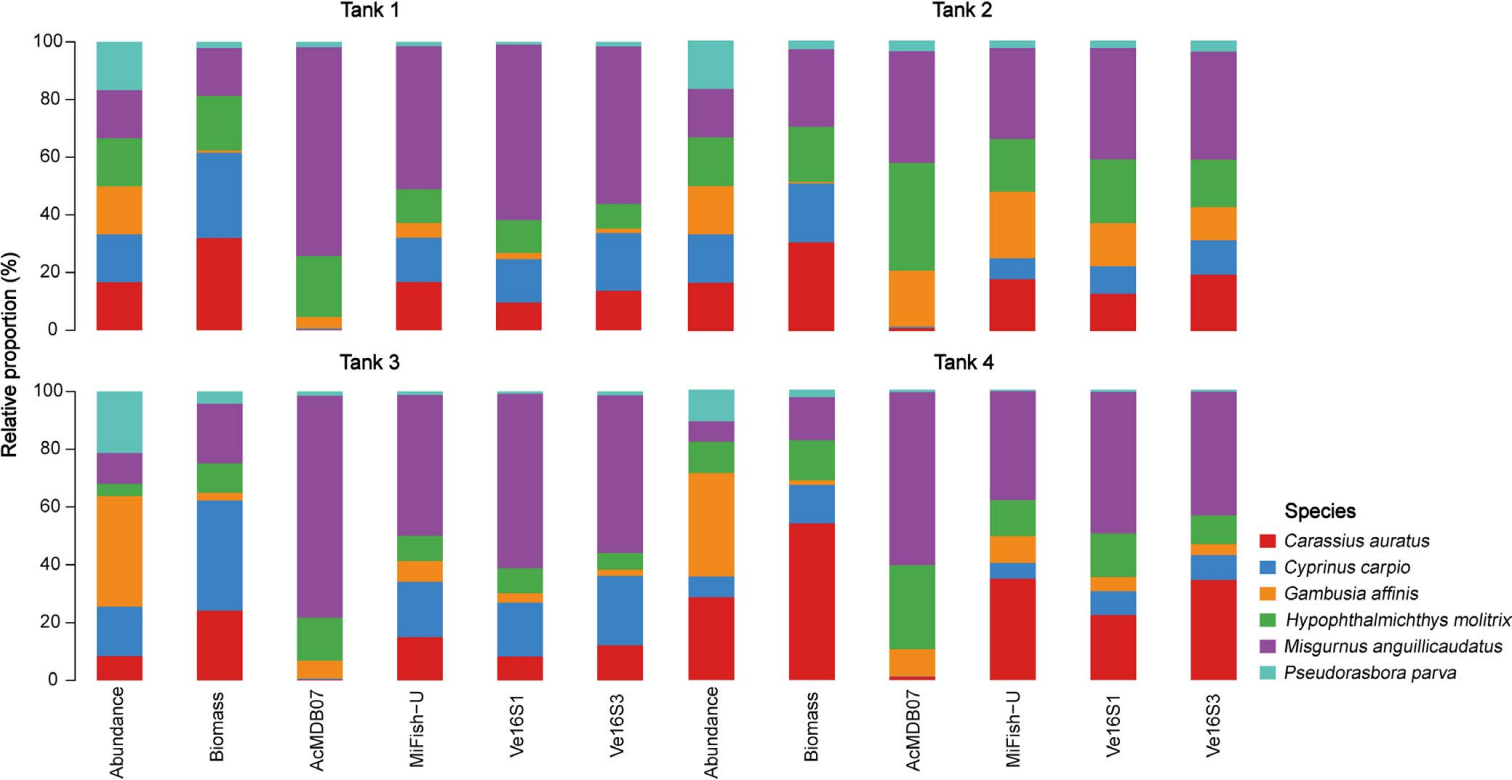
Bar charts showing relative individual abundance, biomass, and read abundance detected using the four primer pairs for each species in each tank. Different colors represent the six species: red (*Carassius auratus*); blue (*Cyprinus carpio*); orange (*Gambusia affinis*); green (*Hypophthalmichthys molitrix*); purple (*Misgurnus anguillicaudatus*); cyan (*Pseudorasbora parva*)

**FIGURE 3:**
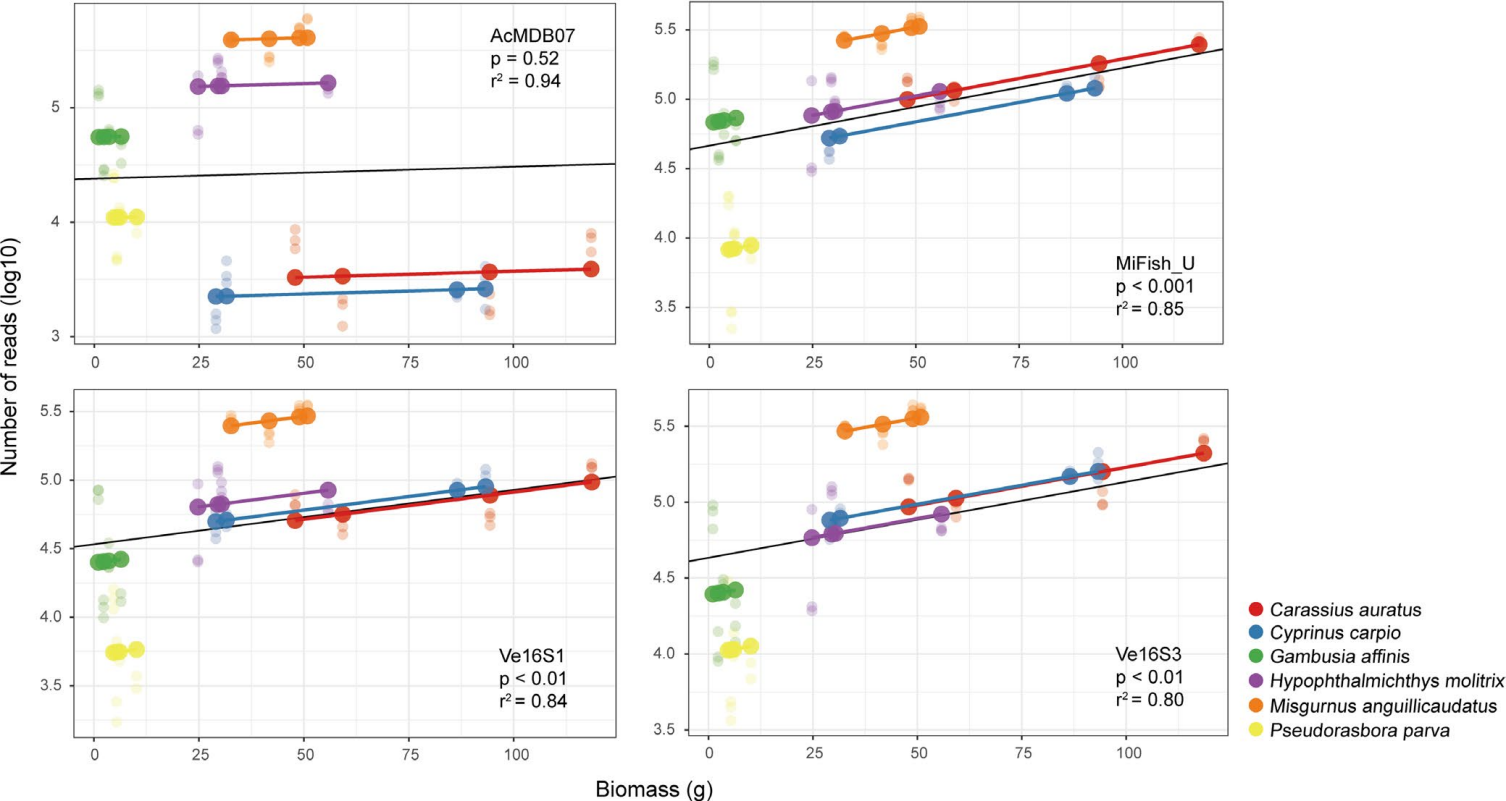
Linear regressions of biomasses (g) and read counts (log10‐transformed) of all species combined within the four tanks detected using each of the four primer pairs. Linear regression analysis results in fitting the linear mixed‐effects (LME) model to account for the influences of biomasses (i.e., fixed effects) and species (i.e., random effects) on the sequence abundances. Data points in different colors represent the six species: red (*Carassius auratus*); blue (*Cyprinus carpio*); green (*Gambusia affinis*); purple (*Hypophthalmichthys molitrix*); orange (*Misgurnus anguillicaudatus*); and yellow (*Pseudorasbora parva*). Significant positive relationships between species biomass and read count were found using the MiFish‐U (*p* < .001, *r* ^2^ = 0.85), Ve16S1 (*p* < .01, *r* ^2^ = 0.84), and Ve16S3 (*p* < .01, *r* ^2^ = 0.80) primer pairs. Data pooled from all tanks (*n* = 72)

All four primer pairs accurately detected the six fish species of each tank, which had varying abundances and biomass; however, the accuracy of species abundance or biomass estimation based on relative read abundance varied with primer selection (Figure 2). The number of reads for each species detected per tank differed among the four primer pairs, even for Tanks 1 and 2, each of which had the same number of individuals for each species (Figure 2). No significant linear relationships between species abundance and read count (Figure S3) were found in the pooled data from all tanks detected using the four primer pairs. However, a significant positive relationship between species biomass and read count was found for MiFish‐U (*p* < .001, *r* ^2^ = 0.85), Ve16S1 (*p* < .01, *r* ^2^ = 0.84), and Ve16S3 (*p* < .01, *r* ^2^ = 0.80) (Figure 3), revealing their potential for metabarcoding quantification of fish biomass. Among the four primer pairs, species‐specific data from the four tanks did not exhibit significant linear relationships between species biomass and read count for most species (Figures [Link], [Link], [Link], [Link]). Only *C. carpio* illustrated a significant positive relationship with a similar *r* ^2^ detected using MiFish‐U (*p* < .05, *r* ^2^ = 0.95), Ve16S1 (*p* <.001, *r* ^2^ = 0.80), and Ve16S3 (*p* <.05, *r* ^2^ = 0.93) (Figures [Link], [Link], [Link]). No significant linear relationships between species biomass and read count were found in the individual tank data detected using the four primer pairs (Figures [Link], [Link], [Link], [Link]).

### 3.4 Amplification differences

**FIGURE 4:**
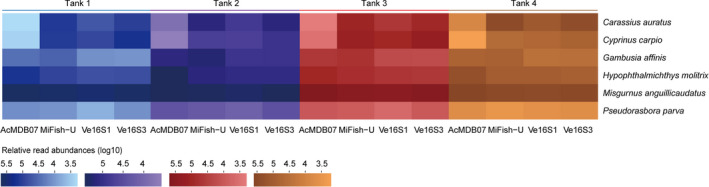
Heatmap showing the relative read abundances for each species of each tank detected using the four primer pairs. All data matrices were transformed by log10

**FIGURE 5:**
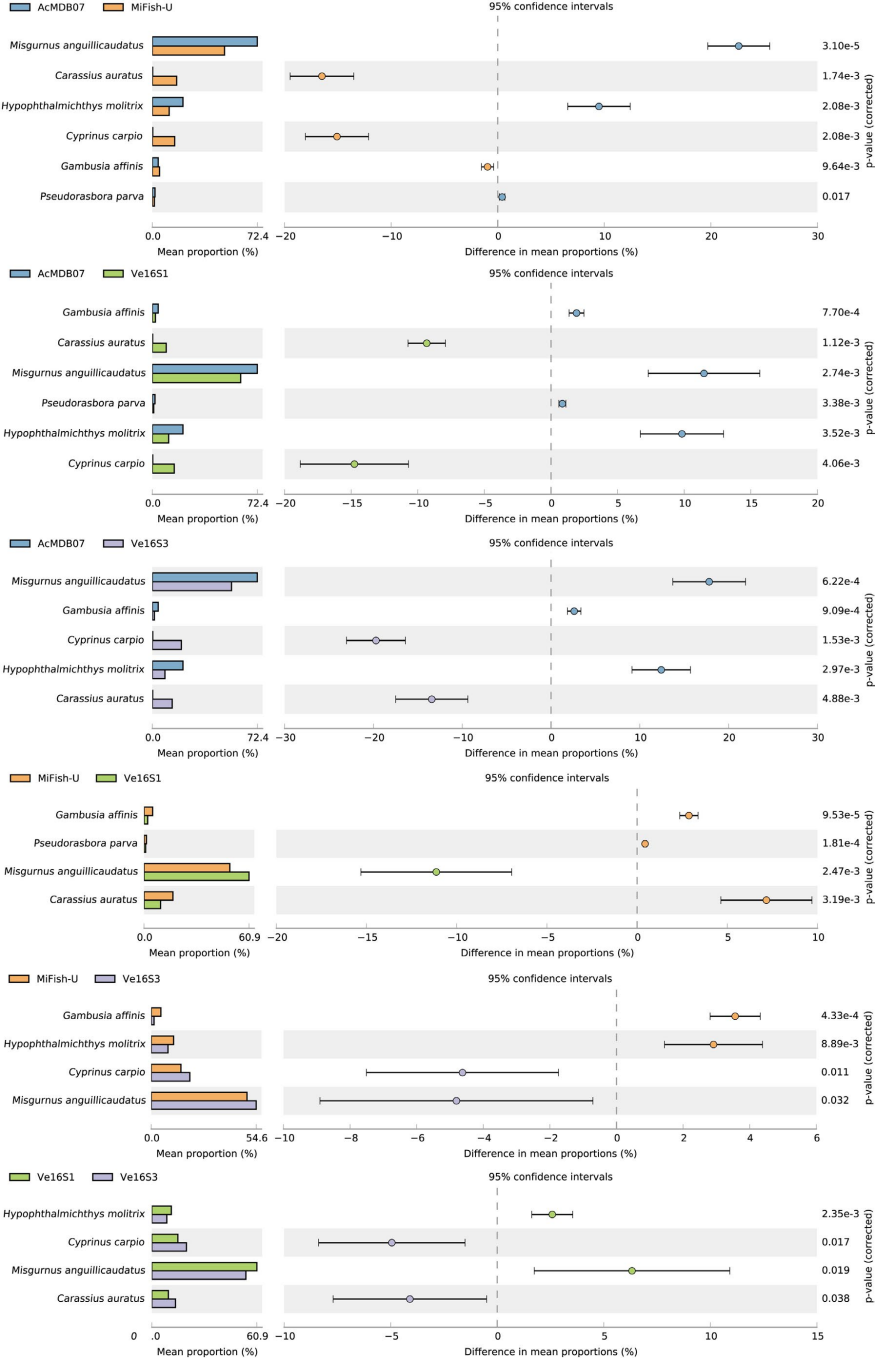
Comparisons of the number of reads per species between primer pairs for Tank 1 (see Figures [Link], [Link], [Link] for additional tanks) were performed using STAMP software v2.1.3. Boxplots were plotted only when significant differences (*p* < .05) in species reads were determined between two primer pairs

Differences in relative read abundance for each species per tank detected by the four primer pairs are visualized by the heatmap in Figure 4. The relative read abundances for *M. anguillicaudatus* and *P. parva* per tank detected by the four primer pairs appeared to be similar, whereas those of *C. auratus*, *C*. *carpio*, *G. affinis,* and *H. molitrix* were highly variable among the four tanks. The relative read abundances for *C. auratus* and *C. carpio* detected by AcMDB07 were lower than detected by MiFish‐U, Ve16S1, and Ve16S3. The relative read abundances for *G. affinis* detected by AcMDB07 and MiFish‐U were higher than detected by Ve16S1 and Ve16S3. The relative read abundances for *H. molitrix* detected by AcMDB07 were higher than detected by MiFish‐U, Ve16S1, and Ve16S3. Primer pair comparisons between the number of reads per species for each tank are shown in Figure 5 and Figures [Link], [Link], [Link]. Species with significant differences of read numbers between two primer pairs varied among the four tanks without a consistent pattern.

## 4 DISCUSSION

An ideal metabarcoding primer pair should have a short amplification length, high taxonomic coverage, unambiguous taxonomic resolution, high primer specificity, and unbiased amplification of the target organism (Bylemans et al., 2018; Collins et al., 2019; Taberlet et al., 2018). However, our in silico primer evaluation revealed that this is an unrealistic goal. From a total of 18 primers, AcMDB07, MiFish‐U, Ve16S1, and Ve16S3 could amplify a large proportion of the fish species in the MitoFish database (79.49%–91.12%) and offer appropriate amplification length (218–321 bp). However, the four primer pairs exhibited different advantages and disadvantages. MiFish‐U had the shortest amplification length of the four primer pairs, but its discriminatory ability for each species was much lower than that of AcMDB07, while AcMDB07 had the highest taxonomic resolution as well as the longest amplification length of the four primer pairs. In practice, short barcode lengths increase species detection by recovering highly degraded eDNA in environmental samples, but show lower taxonomic resolution due to possession of fewer genetic information sites (Bylemans et al., 2018; Jo et al., 2017). Therefore, when selecting optimal primers for eDNA metabarcoding, the aforementioned criteria need to be considered individually. The amplification biases of the four primer pairs that passed the initial screening did not differ using the MitoFish database, indicating their applicability for further tank tests.

The tank experiments aimed to assess for each primer pair: (a) detection ability of species diversity; (b) performance of multispecies eDNA quantification; and (c) species‐level amplification bias. The tank metabarcoding results indicated that the four primer pairs were able to detect all six fish species present in each of the tanks, regardless of even/skewed abundance and high/low biomass. Previous studies have indicated that eDNA metabarcoding is less likely to detect rare species (Evans et al., 2015; Valentini et al., 2016). One reason for the detection bias is that some primers may preferentially amplify high‐abundance species, an amplification bias that could be magnified by HTS (Elbrecht & Leese, 2015). For this reason, using multiple loci was recommended to reduce primer bias. The four primer pairs in the current study showed unbiased detection of the full species diversity in the varying fish assemblages of the four tanks, which was consistent with their performance in previous studies (Cilleros et al., 2019; Nakagawa et al., 2018; Simmons et al., 2016; Vences et al., 2016). However, this does not mean that the four primer pairs did not have amplification biases for the target species that hindered the accuracy of their quantification.

A significant positive relationship was observed between species biomass and read count using the pooled tank data detected by the MiFish‐U, Ve16S1, and Ve16S3 primer pairs. A positive relationship between taxa relative abundance or biomass and taxa sequence counts was also demonstrated in previous studies (Evans et al., 2015; Hanfling et al., 2016; Klobucar et al., 2017; Thomsen et al., 2016; Ushio et al., 2018), which supported the potential of using metabarcoding sequencing data for multispecies quantitative estimations. However, our results indicated that positive relationships were not present for all species when analyzing species‐specific data. Similarly, such positive relationships were not found when analyzing individual tank data. In our experimental tanks, species with the lowest and highest biomass were not always detected with the lowest and highest numbers of reads, respectively. It is difficult to account for differences in eDNA production among species affected by physiological traits, activity level, and metabolism, or the confounding influences of technical process biases on the disconnect between read count and eDNA concentration in the controlled tanks, let alone in complex natural ecosystems (Evans et al., 2015; Hanfling et al., 2016; Kelly et al., 2014; Knudsen et al., 2019; Shaw et al., 2016). Sequence read counts are not a reliable index for fish abundance/biomass, as they are influenced by biases from various sources, including sampling strategy, laboratory process, and analysis (amplification, sequencing, and bioinformatics), as well as the origin and fate of species eDNA in different environments (Evans & Lamberti, 2018; Lacoursiere‐Roussel & Deiner, 2019; Shaw et al., 2016; Ushio et al., 2018). Ushio et al. (2018) recommended the addition of internal standard DNA (i.e., known copy number of short DNA fragments from nontarget species) to eDNA samples to determine the presence of biases within metabarcoding data for eDNA quantitative analysis of fish. Our analysis of the relationship between read counts and species abundance or biomass indicated that read abundance was strongly associated with species biomass rather than species abundance, which has been demonstrated previously by Evans et al. (2015). In controlled experiments, eDNA production was also positively associated with biomass/density in bluegill sunfish (Maruyama et al., 2014) and common carp (Doi et al., 2015). Furthermore, it would be challenging to assess the abundance of a particular species in a natural habitat if the individuals are at different developmental stages, and, therefore, different biomass. Consequently, organism biomass rather than number of individuals appears to strongly influence the amount of DNA that organisms release into the water.

Species‐level amplification difference analyses illustrated that the amplification efficiency of each primer pair varied depending on the species, and the preferential species varied depending on the fish assemblage, of which both findings were also demonstrated in previous studies (Bellemain et al., 2010; Elbrecht & Leese, 2015). Such complex situations are unable to exclude other confounding influences, such as species traits, experimental processes, and analysis methods. Our results illustrated the uncertainty of multispecies quantitative estimation by eDNA metabarcoding even under controlled conditions.

## 5 CONCLUSIONS

Environmental DNA metabarcoding has great potential in species detection and biodiversity assessment by providing qualitative information concerning the presence/absence of species and ranking sequence reads for species abundance and diversity. However, due to lack of reliable evidence supporting a positive relationship between relative species and sequence abundances, eDNA metabarcoding is not currently recommended for multispecies quantitative estimation. Our results indicated that the four primer pairs demonstrated good qualitative ability for species detection in the controlled tanks with even or skewed relative species abundance. The positive relationship between species biomass and read count for the pooled tank data indicated the potential of estimating relative species biomass in the identified communities using metabarcoding sequencing data. Unfortunately, this positive relationship was not found for all species and primer efficiency differed depending on the species. Even under controlled tank conditions, it is difficult to account for confounding influences that result in eDNA quantitative estimation bias. Thus, quantitative estimates based on taxon sequence abundance via eDNA metabarcoding may not be realistic.

Our study provides an alternative strategy for evaluating the performance of available primers in silico and in controlled tanks to select suitable primers for taxon groups of interest. Local species biodiversity information (e.g., previous results via traditional monitoring) and primer testing are strongly recommended before beginning work in any ecosystem of interest. Primer selection bias is only one aspect that results in eDNA detection uncertainty, and more research is required to explore how eDNA metabarcoding may be used to obtain reliable data for biodiversity monitoring. Our findings emphasize that eDNA cannot replace classical monitoring, particularly if population size data are needed for fish conservation and management. It remains necessary to combine traditional capture‐based methods with eDNA metabarcoding when attempting to quantify the species richness of a natural community to avoid biased quantitative estimates.

## CONFLICT OF INTEREST

The authors declare no conflict of interest.

## AUTHOR CONTRIBUTIONS

**Lu Shu:** Conceptualization (equal); Formal analysis (lead); Investigation (lead); Writing‐original draft (lead); Writing‐review & editing (supporting). **Arne Ludwig:** Conceptualization (equal); Formal analysis (equal); Writing‐original draft (supporting); Writing‐review & editing (equal). **Zuogang Peng:** Conceptualization (lead); Formal analysis (equal); Project administration (lead); Supervision (lead); Writing‐original draft (supporting); Writing‐review & editing (equal).

## Supporting information

Figure S1

Figure S2

Figure S3

Figure S4

Figure S5

Figure S6

Figure S7

Figure S8

Figure S9

Figure S10

Figure S11

Figure S12

Figure S13

Figure S14

Table S1‐S3

## Data Availability

The raw sequence data are available on the NCBI Sequence Read Archive (SRA) database (Accession Number: SRR13567471‐SRR13567518). All other datasets supporting this study are available in the article and its supporting information.
