# Supplementary figures and images for: Environmental DNA metabarcoding primers for freshwater fish detection and quantification: In silico and in tanks
Source: Ecol Evol. 2021 May 16;11(12):8281–94. doi: 10.1002/ece3.7658 (PMC8216916; doi:10.1002/ece3.7658)

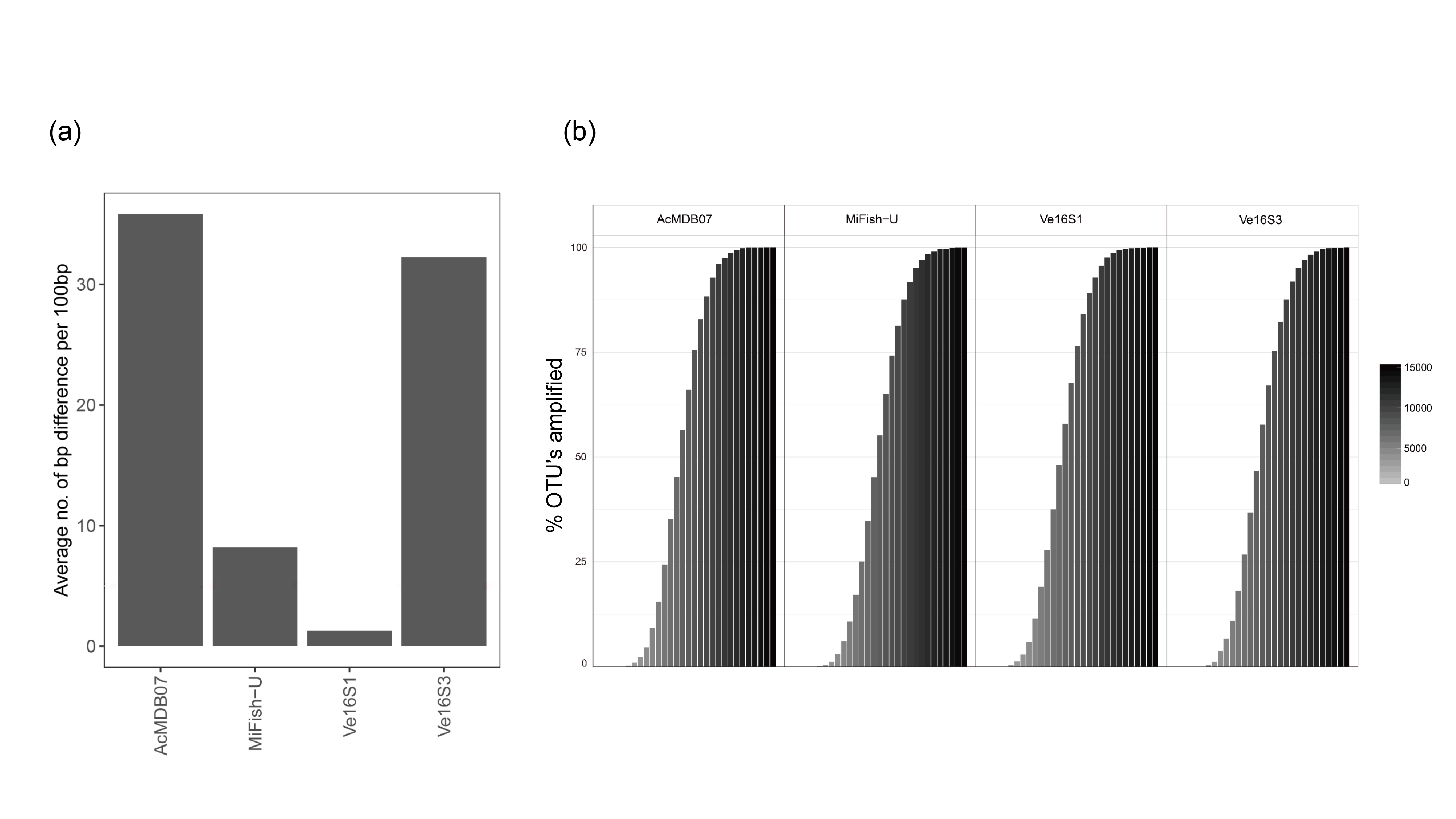

Supplement: Supplementary file 1 — Figure S1 [file ECE3-11-8281-s001.jpg]

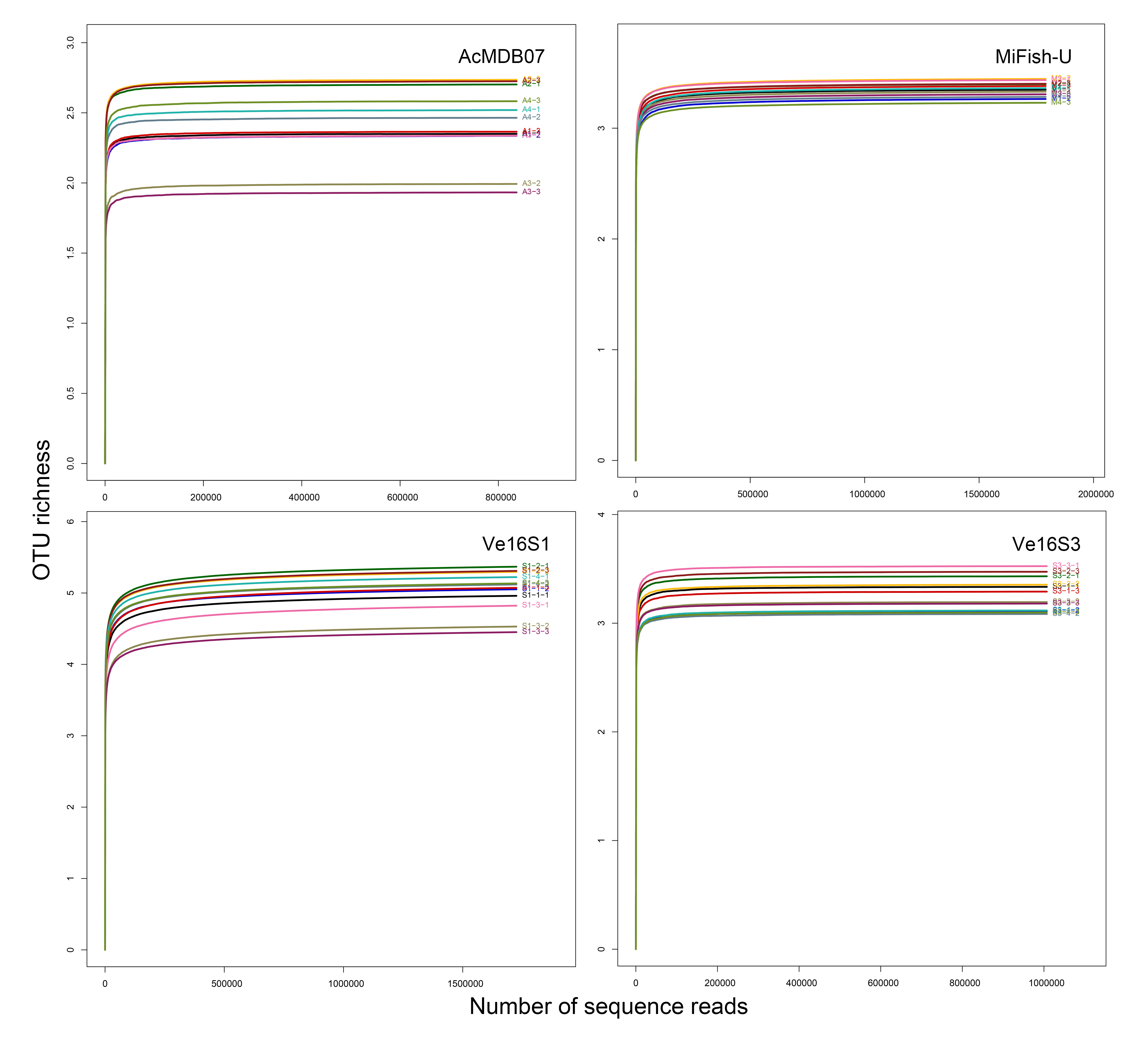

Supplement: Supplementary file 2 — Figure S2 [file ECE3-11-8281-s006.jpg]

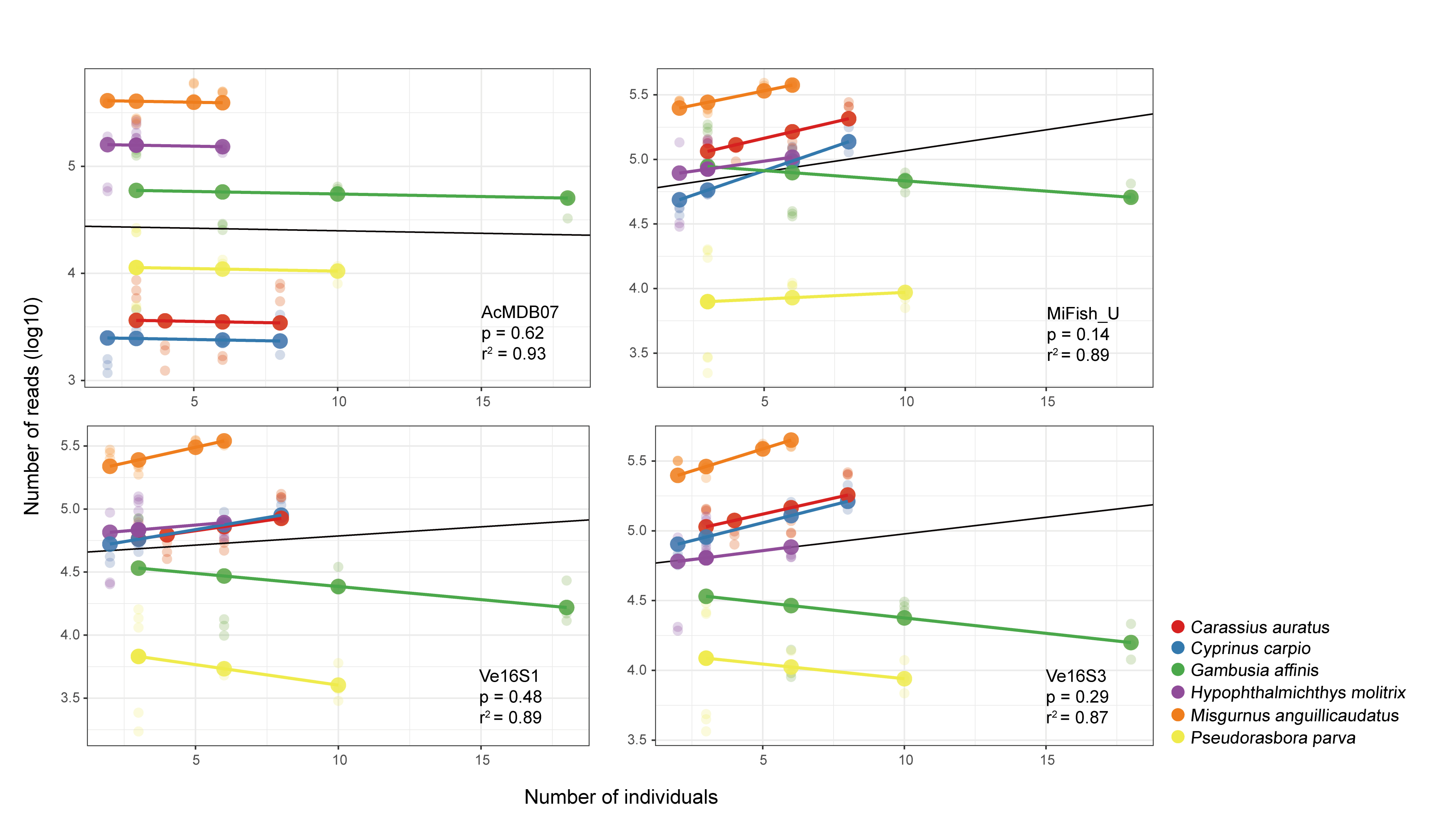

Supplement: Supplementary file 3 — Figure S3 [file ECE3-11-8281-s003.jpg]

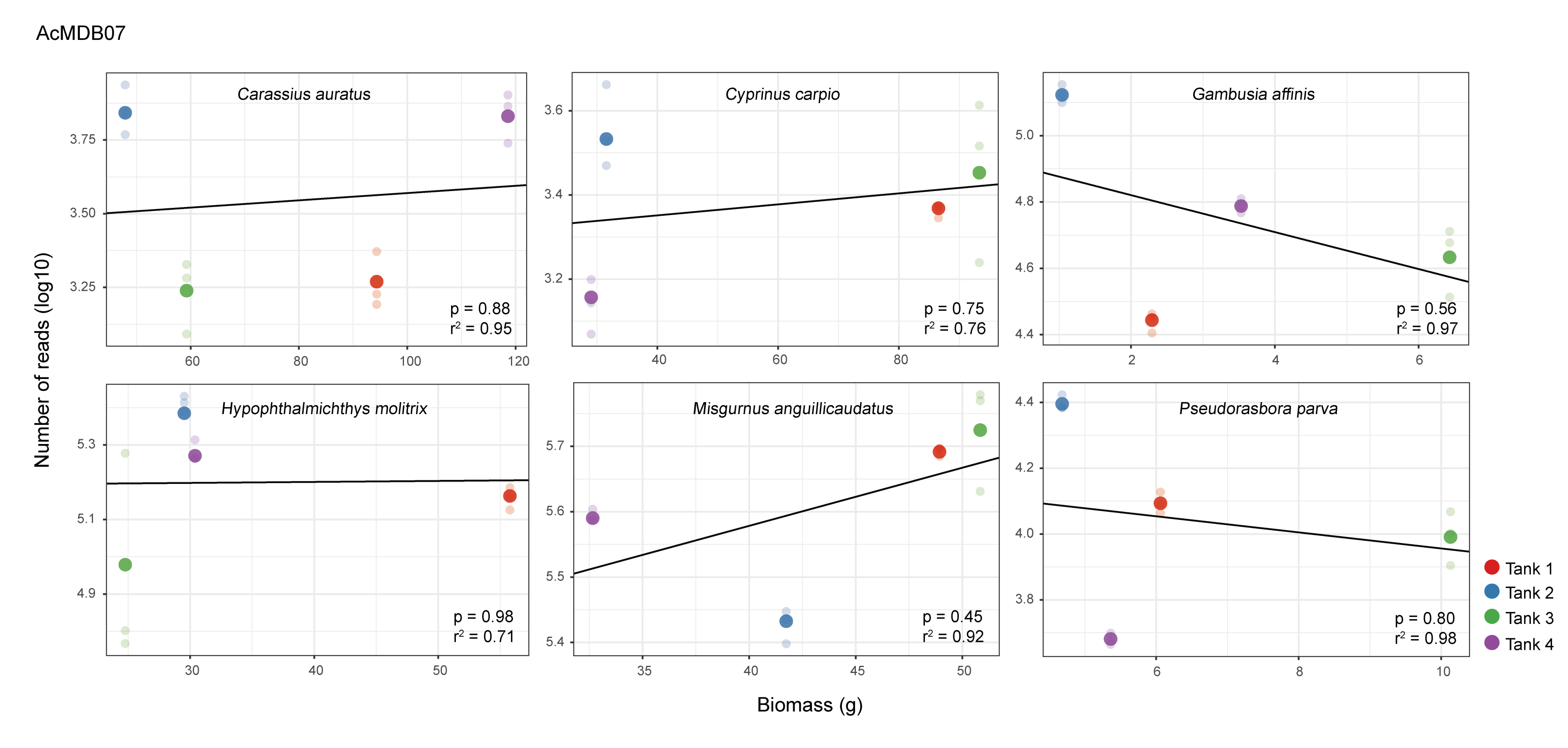

Supplement: Supplementary file 4 — Figure S4 [file ECE3-11-8281-s008.jpg]

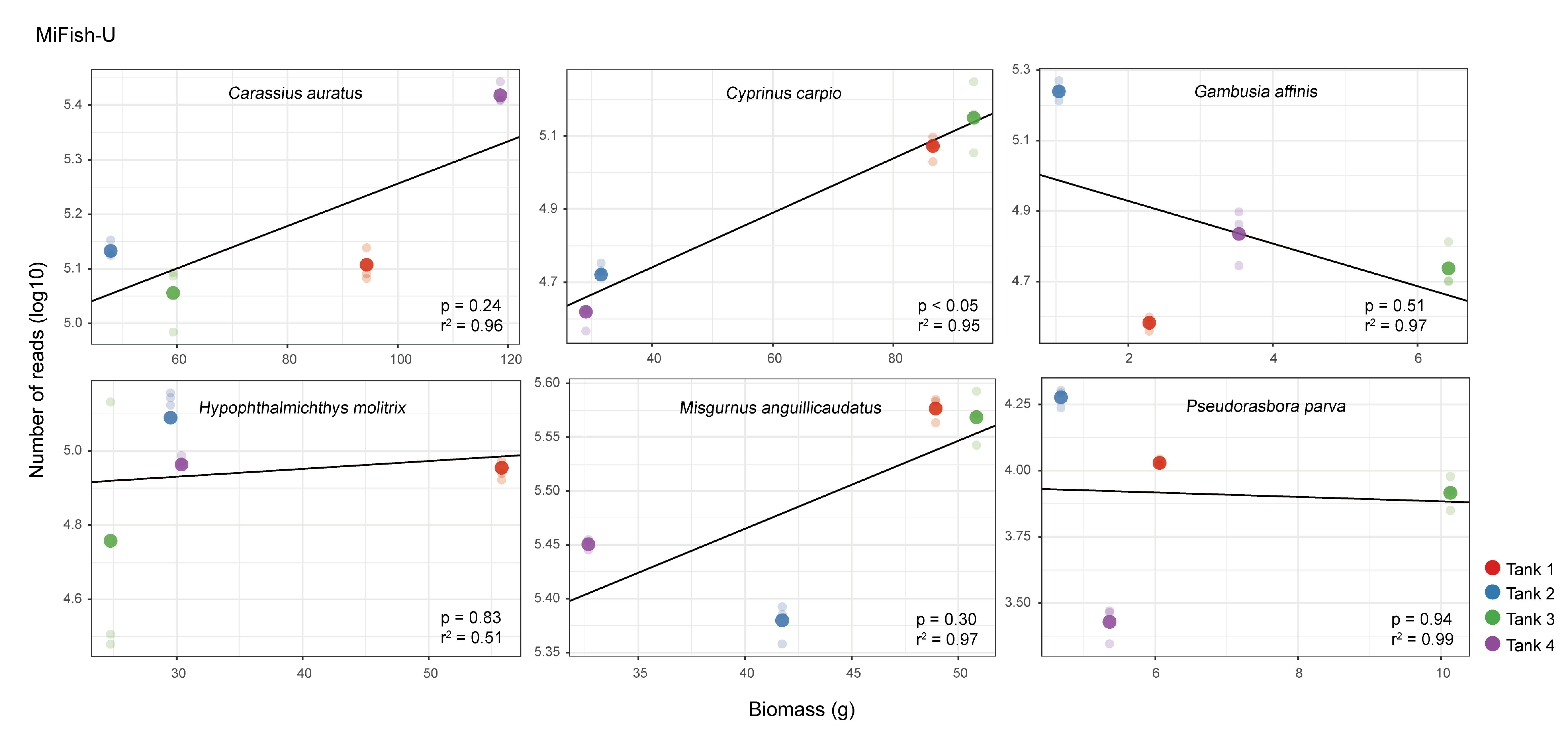

Supplement: Supplementary file 5 — Figure S5 [file ECE3-11-8281-s014.jpg]

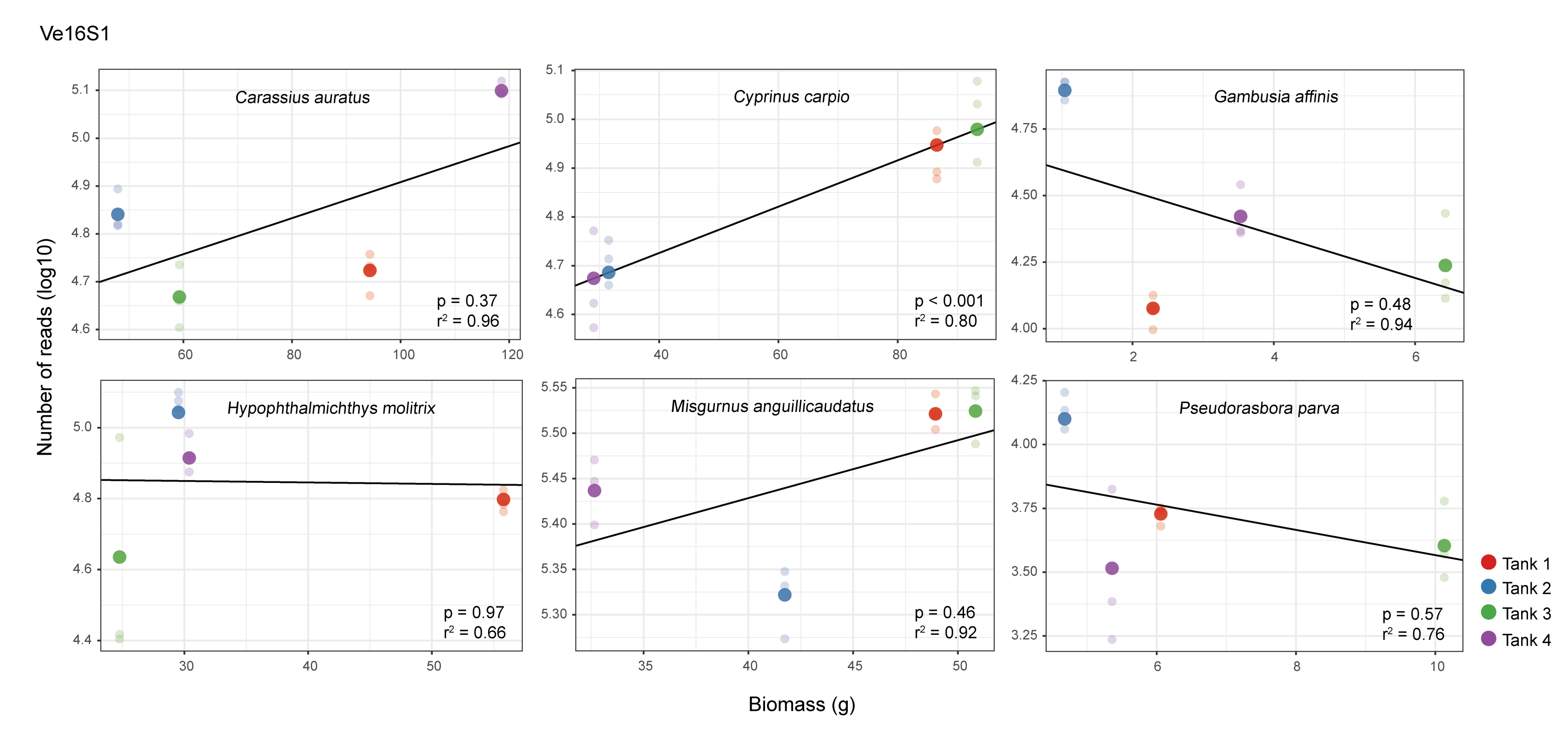

Supplement: Supplementary file 6 — Figure S6 [file ECE3-11-8281-s009.jpg]

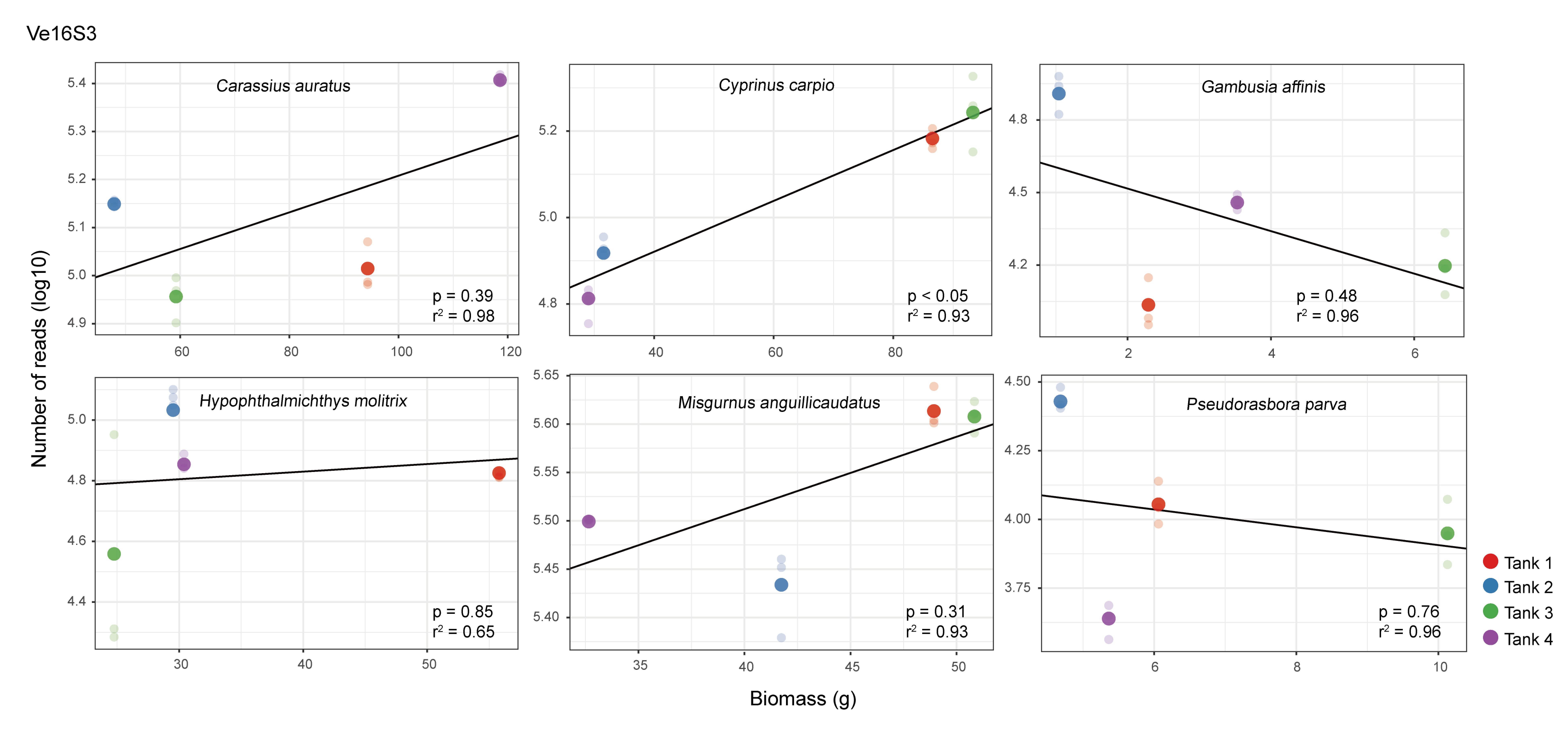

Supplement: Supplementary file 7 — Figure S7 [file ECE3-11-8281-s015.jpg]

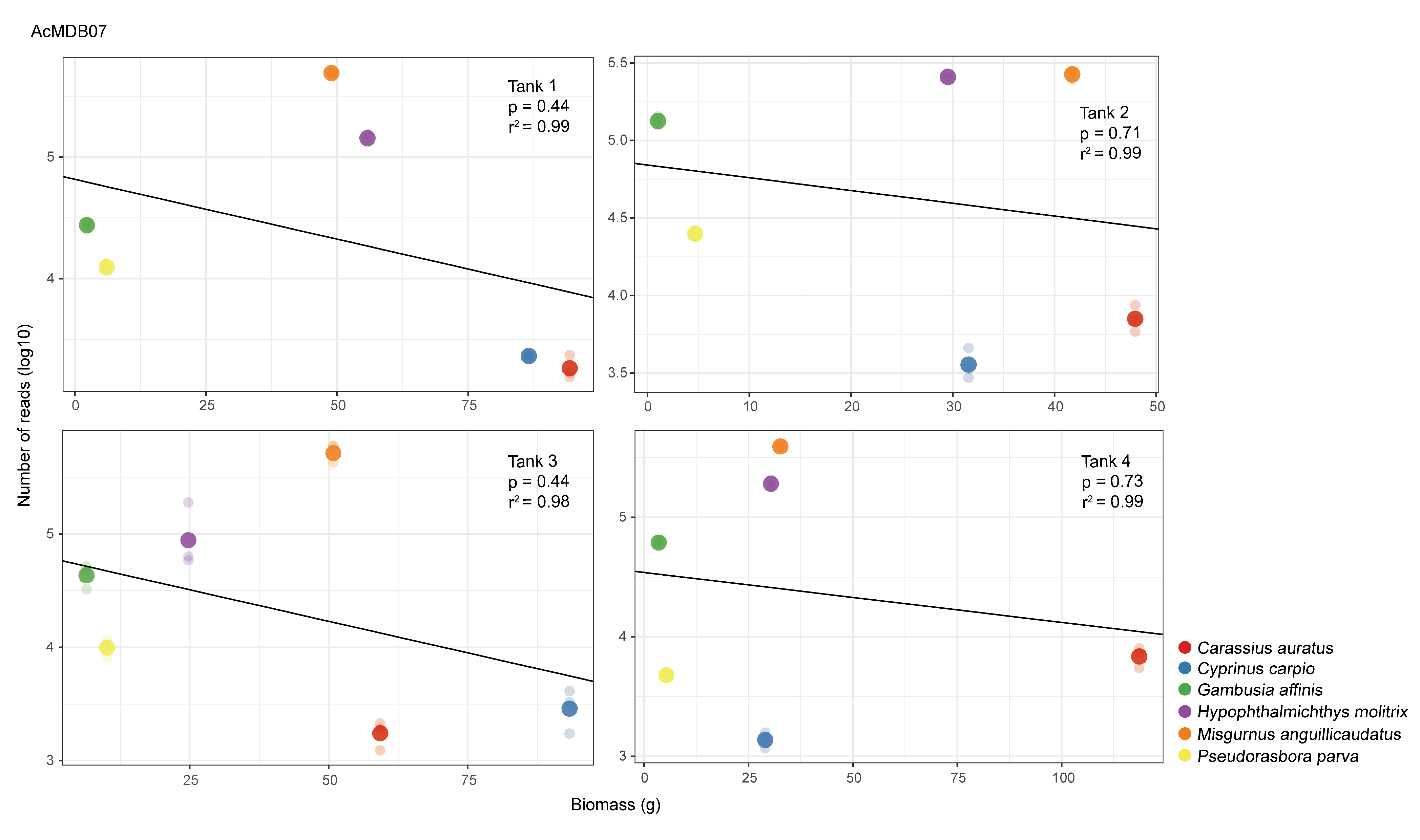

Supplement: Supplementary file 8 — Figure S8 [file ECE3-11-8281-s012.jpg]

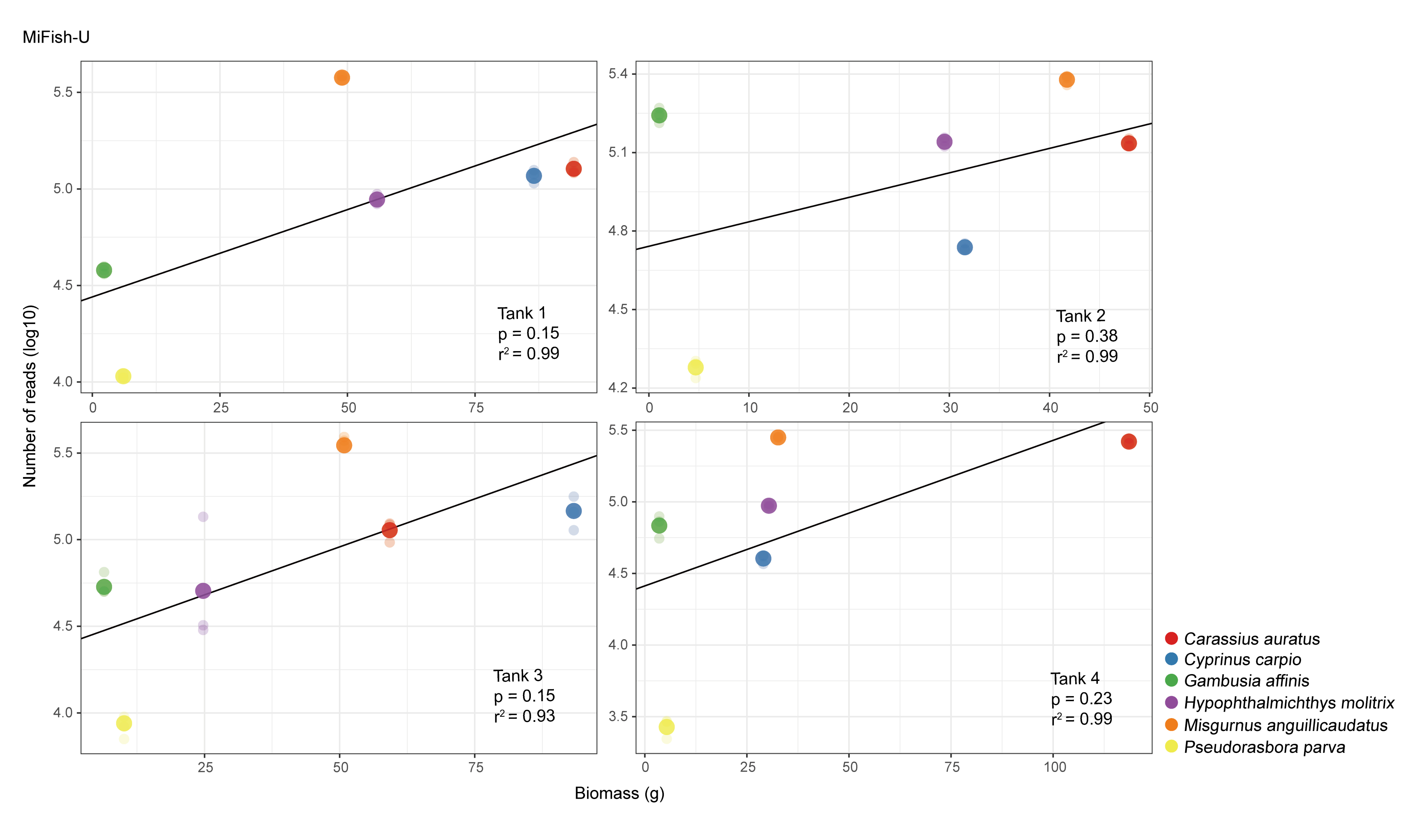

Supplement: Supplementary file 9 — Figure S9 [file ECE3-11-8281-s011.jpg]

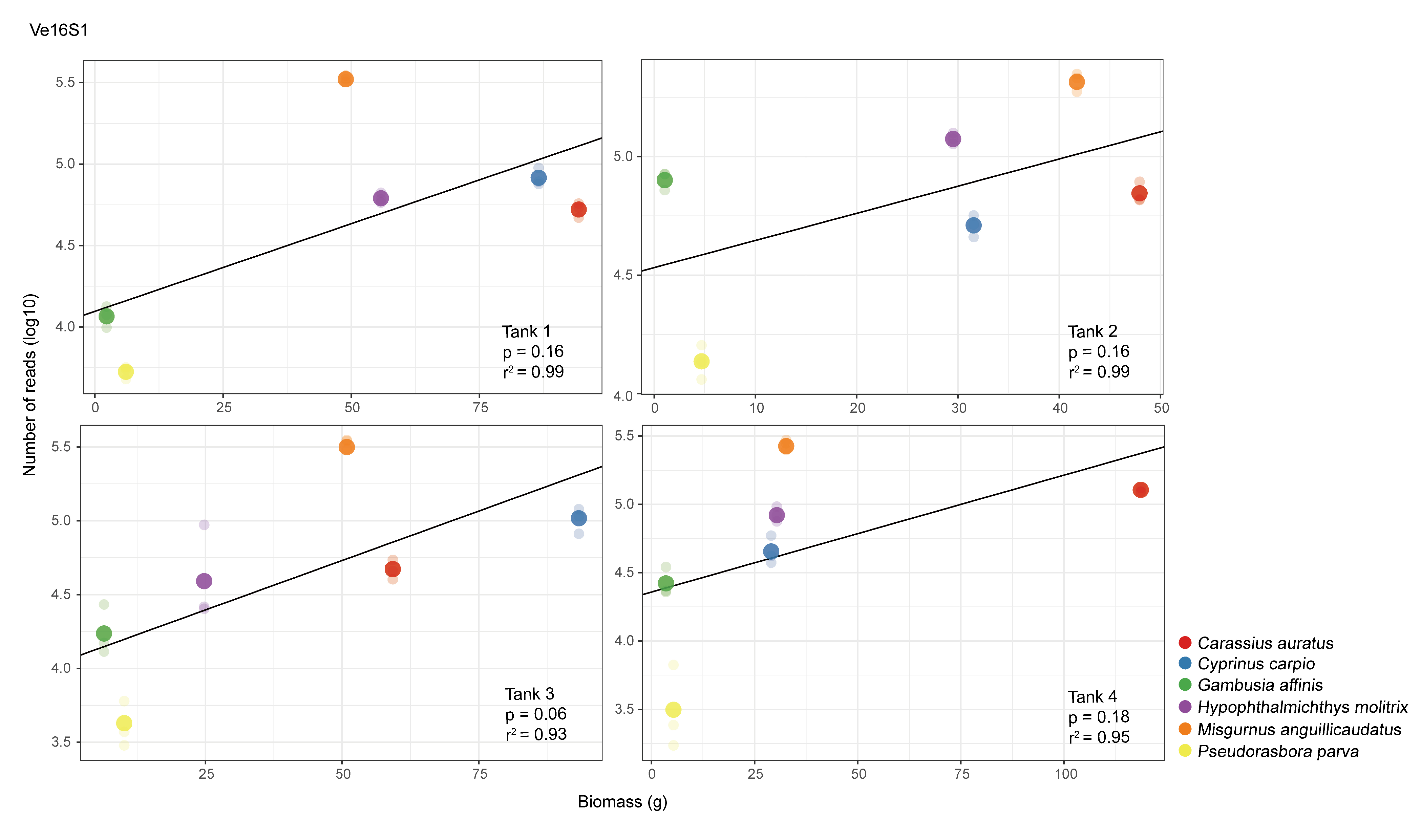

Supplement: Supplementary file 10 — Figure S10 [file ECE3-11-8281-s002.jpg]

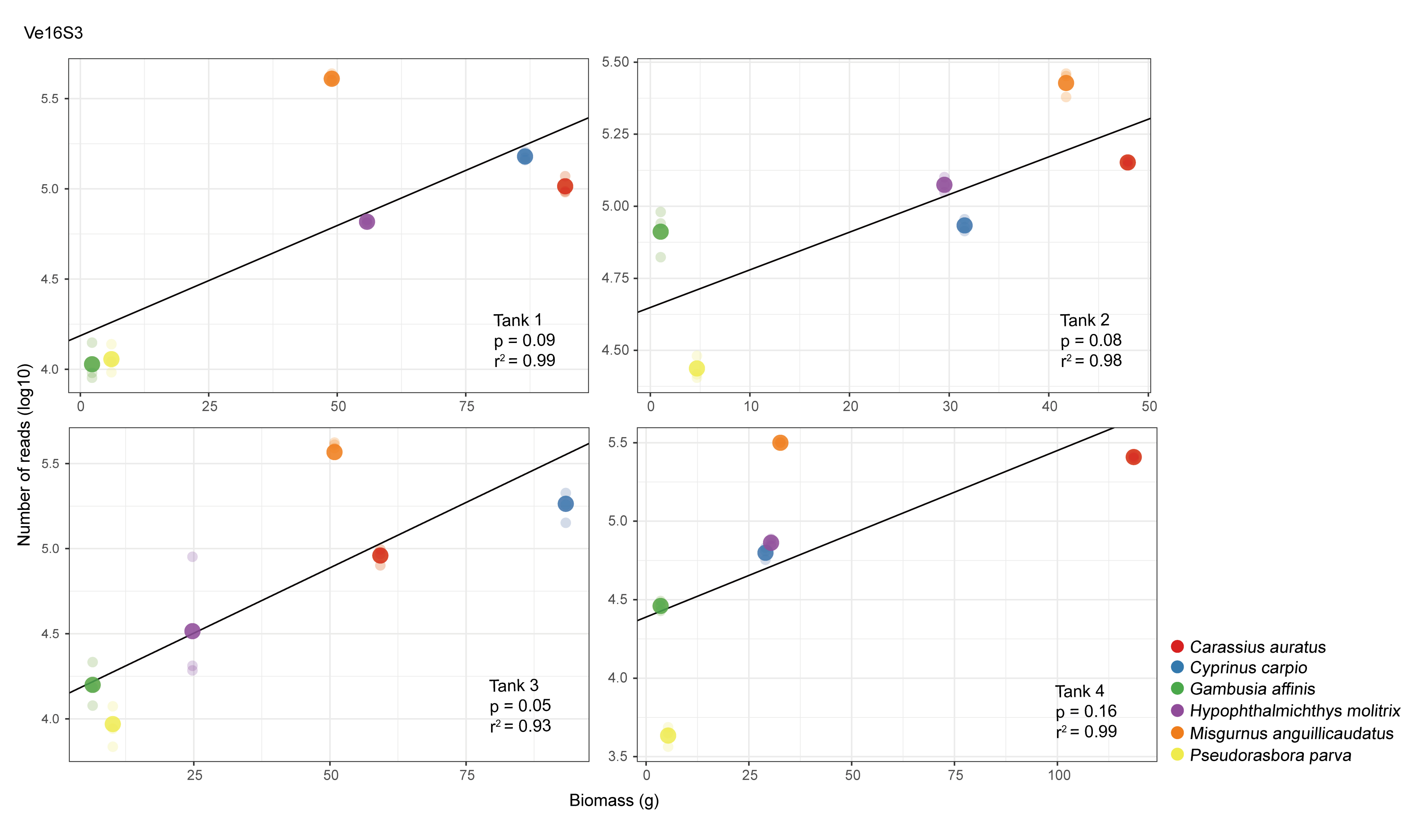

Supplement: Supplementary file 11 — Figure S11 [file ECE3-11-8281-s007.jpg]

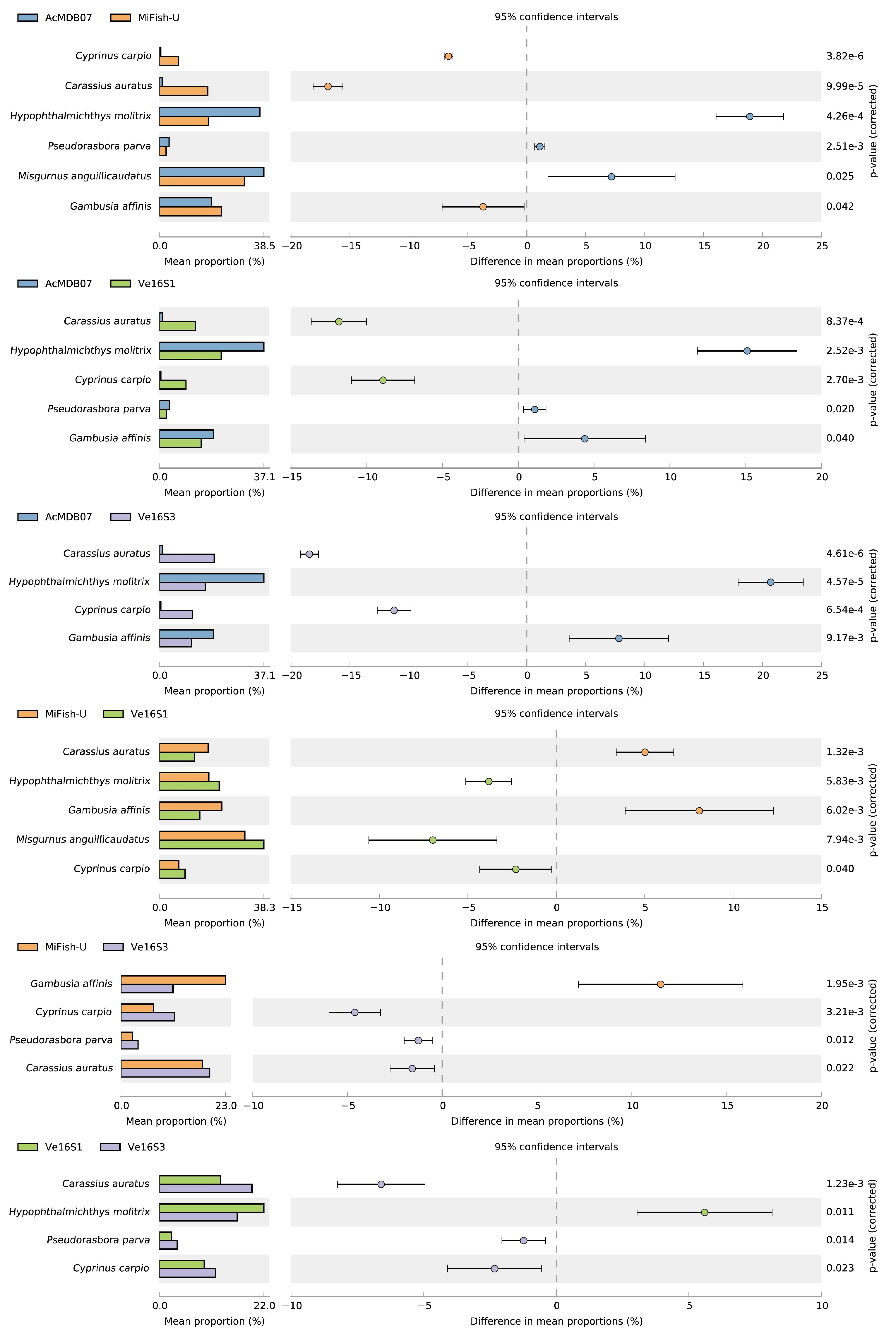

Supplement: Supplementary file 12 — Figure S12 [file ECE3-11-8281-s010.jpg]

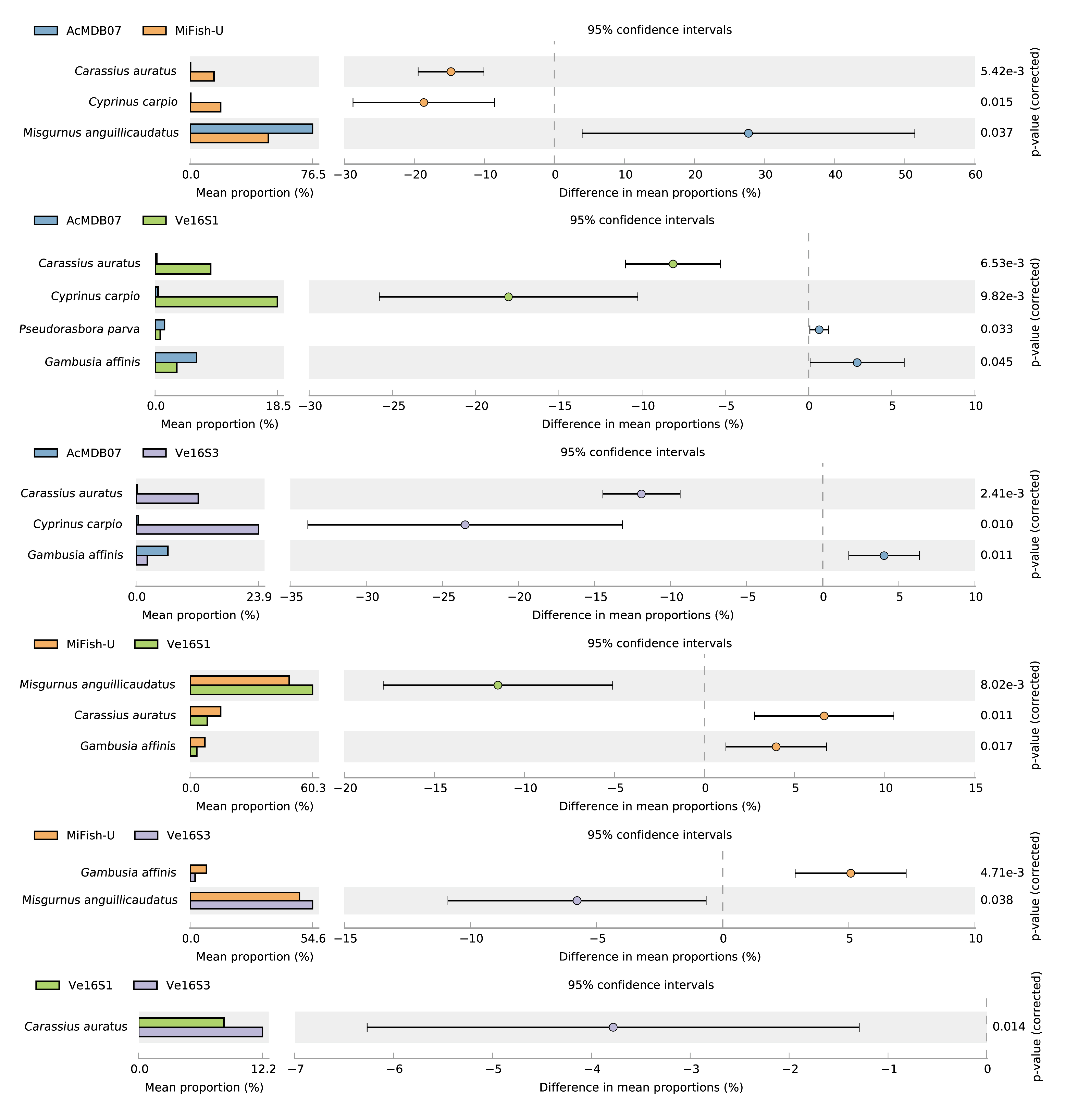

Supplement: Supplementary file 13 — Figure S13 [file ECE3-11-8281-s004.jpg]

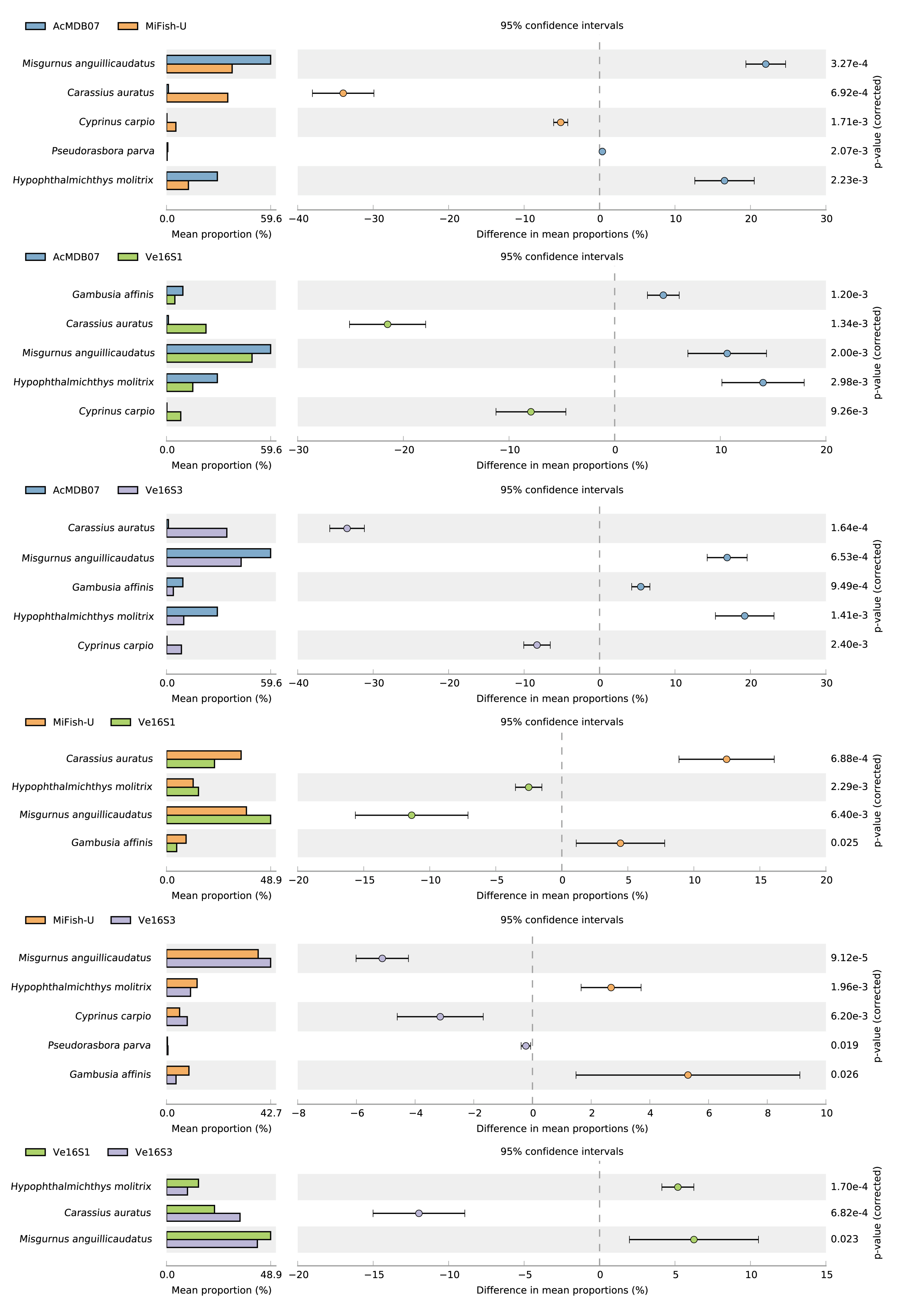

Supplement: Supplementary file 14 — Figure S14 [file ECE3-11-8281-s005.jpg]
