# Supplementary material for: Environmental DNA metabarcoding primers for freshwater fish detection and quantification: In silico and in tanks
Source: Ecol Evol. 2021 May 16;11(12):8281–94. doi: 10.1002/ece3.7658 (PMC8216916; doi:10.1002/ece3.7658)
Supplement: Supplementary file 15 — Table S1‐S3 [file ECE3-11-8281-s013.docx]

| Table S1. Total mortality (number of individuals) of each species during the 5-day tank experiment. | | | | | | | | | | | | | | | | | | | | |
| --- | --- | --- | --- | --- | --- | --- | --- | --- | --- | --- | --- | --- | --- | --- | --- | --- | --- | --- | --- | --- |
| **Species** | **High density, even abundance** | | | | | **Low density, even abundance** | | | | | **High density, skewed abundance** | | | | | **Low density, skewed abundance** | | | | |
|  | **Tank 1 (100 L)** | | | | | **Tank 2 (100 L)** | | | | | **Tank 3 (100 L)** | | | | | **Tank 4 (100 L)** | | | | |
|  | Day 1 | Day 2 | Day 3 | Day 4 | Day 5 | Day 1 | Day 2 | Day 3 | Day 4 | Day 5 | Day 1 | Day 2 | Day 3 | Day 4 | Day 5 | Day 1 | Day 2 | Day 3 | Day 4 | Day 5 |
| *Carassius auratus* | 2 |  |  |  |  |  |  |  |  |  | 2 |  |  |  |  |  | 1 |  |  |  |
| *Cyprinus carpio* |  |  |  |  |  |  |  |  |  |  |  |  |  |  |  | 1 |  |  |  |  |
| *Hypophthalmichthys molitrix* |  |  |  |  |  |  |  |  |  |  |  |  |  |  |  |  |  |  |  |  |
| *Misgurnus anguillicaudatus* |  | 1 |  |  |  |  |  |  |  |  |  | 1 | 1 | 1 |  |  |  |  |  |  |
| *Pseudorasbora parva* | 1 |  |  | 1 |  |  |  |  |  |  | 1 |  |  |  |  |  |  |  |  |  |
| *Gambusia affinis* | 2 |  | 1 |  | 1 | 1 |  |  |  | 1 | 6 |  |  |  | 3 | 3 | 1 |  |  |  |

| Table S2a. Standardized reads for each species at each tank detected with the AcMDB07 primers in total 12 replicate samples. | | | | | | | | | | | | | |
| --- | --- | --- | --- | --- | --- | --- | --- | --- | --- | --- | --- | --- | --- |
| Species | High density, even abundance | | | Low density, even abundance | | | High density, skewed abundance | | | Low density, skewed abundance | | |  |
|  | Tank 1 | | | Tank 2 | | | Tank 3 | | | Tank 4 | | |  |
|  | Sample 1-1 | Sample 1-2 | Sample 1-3 | Sample 2-1 | Sample 2-2 | Sample 2-3 | Sample 3-1 | Sample 3-2 | Sample 3-3 | Sample 4-1 | Sample 4-2 | Sample 4-3 |  |
| *Carassius auratus* | 1558 | 1688 | 2352 | 5866 | 8646 | 6933 | 1235 | 1913 | 2127 | 7993 | 5483 | 7314 |  |
| *Cyprinus carpio* | 2342 | 2215 | 2373 | 3379 | 4591 | 2949 | 1735 | 4103 | 3282 | 1392 | 1173 | 1581 |  |
| *Gambusia affinis* | 25413 | 29023 | 28314 | 125830 | 142528 | 132746 | 32610 | 47551 | 51451 | 61416 | 58515 | 64522 |  |
| *Hypophthalmichthys molitrix* | 153310 | 144844 | 133531 | 258835 | 269455 | 244385 | 189504 | 63377 | 58526 | 183315 | 205892 | 184388 |  |
| *Misgurnus anguillicaudatus* | 484184 | 497401 | 497668 | 280098 | 250060 | 272627 | 427721 | 588590 | 601277 | 401502 | 388113 | 385661 |  |
| *Pseudorasbora parva* | 12304 | 13415 | 11580 | 24242 | 26511 | 24227 | 8022 | 10042 | 11685 | 4619 | 4997 | 4712 |  |
|  |  |  |  |  |  |  |  |  |  |  | total reads： | 8,204,762 |  |
|  |  |  |  |  |  |  |  |  |  |  |  |  |  |
| Table S2b. Standardized reads for each species at each tank detected with the MiFish-U primers in total 12 replicate samples. | | | | | | | | | | | | | |
| Species | High density, even abundance | | | Low density, even abundance | | | High density, skewed abundance | | | Low density, skewed abundance | | |  |
|  | Tank 1 | | | Tank 2 | | | Tank 3 | | | Tank 4 | | |  |
|  | Sample 1-1 | Sample 1-2 | Sample 1-3 | Sample 2-1 | Sample 2-2 | Sample 2-3 | Sample 3-1 | Sample 3-2 | Sample 3-3 | Sample 4-1 | Sample 4-2 | Sample 4-3 |  |
| *Carassius auratus* | 123275 | 120984 | 137594 | 133206 | 134176 | 142208 | 96429 | 123913 | 122133 | 277411 | 256263 | 257031 |  |
| *Cyprinus carpio* | 125094 | 119064 | 107092 | 53941 | 53473 | 56611 | 113393 | 177256 | 144534 | 36894 | 41768 | 42263 |  |
| *Gambusia affinis* | 39707 | 37978 | 36236 | 175565 | 186260 | 163383 | 50096 | 50389 | 64973 | 55562 | 72900 | 79078 |  |
| *Hypophthalmichthys molitrix* | 94078 | 86955 | 83596 | 139207 | 132898 | 143393 | 135494 | 30142 | 32073 | 93486 | 97169 | 91774 |  |
| *Misgurnus anguillicaudatus* | 365895 | 384520 | 382917 | 246872 | 228023 | 242788 | 348775 | 372542 | 391441 | 285089 | 278836 | 283955 |  |
| *Pseudorasbora parva* | 11066 | 10482 | 10566 | 17305 | 20106 | 19667 | 7070 | 8336 | 9504 | 2217 | 2911 | 2951 |  |
|  |  |  |  |  |  |  |  |  |  |  | total reads： | 9,102,232 |  |
|  |  |  |  |  |  |  |  |  |  |  |  |  |  |
| Table S2c. Standardized reads for each species at each tank detected with the Ve16S1 primers in total 12 replicate samples. | | | | | | | | | | | | | |
| Species | High density, even abundance | | | Low density, even abundance | | | High density, skewed abundance | | | Low density, skewed abundance | | |  |
|  | Tank 1 | | | Tank 2 | | | Tank 3 | | | Tank 4 | | |  |
|  | Sample 1-1 | Sample 1-2 | Sample 1-3 | Sample 2-1 | Sample 2-2 | Sample 2-3 | Sample 3-1 | Sample 3-2 | Sample 3-3 | Sample 4-1 | Sample 4-2 | Sample 4-3 |  |
| *Carassius auratus* | 53984 | 46839 | 57175 | 65601 | 66033 | 78298 | 40160 | 54361 | 45659 | 123517 | 131614 | 124302 |  |
| *Cyprinus carpio* | 94731 | 77995 | 75447 | 45732 | 51742 | 56515 | 81649 | 119690 | 107422 | 37405 | 59089 | 41963 |  |
| *Gambusia affinis* | 13354 | 9910 | 11851 | 84286 | 84503 | 72228 | 14876 | 13005 | 27106 | 23309 | 34760 | 22905 |  |
| *Hypophthalmichthys molitrix* | 66711 | 57977 | 60808 | 118994 | 113106 | 125720 | 93811 | 25356 | 26147 | 83016 | 96271 | 75008 |  |
| *Misgurnus anguillicaudatus* | 331374 | 319238 | 349259 | 214595 | 187683 | 222636 | 307738 | 347427 | 352093 | 279823 | 295395 | 250527 |  |
| *Pseudorasbora parva* | 5654 | 4793 | 5501 | 11484 | 13630 | 16014 | 3716 | 3013 | 6004 | 2426 | 6682 | 1722 |  |
|  |  |  |  |  |  |  |  |  |  |  | total reads： | 6,630,368 |  |
|  |  |  |  |  |  |  |  |  |  |  |  |  |  |
| Table S2d. Standardized reads for each species at each tank detected with the Ve16S3 primers in total 12 replicate samples. | | | | | | | | | | | | | |
| Species | High density, even abundance | | | Low density, even abundance | | | High density, skewed abundance | | | Low density, skewed abundance | | |  |
|  | Tank 1 | | | Tank 2 | | | Tank 3 | | | Tank 4 | | |  |
|  | Sample 1-1 | Sample 1-2 | Sample 1-3 | Sample 2-1 | Sample 2-2 | Sample 2-3 | Sample 3-1 | Sample 3-2 | Sample 3-3 | Sample 4-1 | Sample 4-2 | Sample 4-3 |  |
| *Carassius auratus* | 95839 | 96943 | 117586 | 138992 | 143557 | 141900 | 79728 | 93030 | 98969 | 262014 | 251815 | 254860 |  |
| *Cyprinus carpio* | 160718 | 148843 | 144391 | 82020 | 90170 | 84576 | 141846 | 212276 | 181345 | 56791 | 67988 | 64607 |  |
| *Gambusia affinis* | 8979 | 9561 | 14056 | 87139 | 95566 | 66567 | 11958 | 15275 | 21537 | 26831 | 28843 | 31017 |  |
| *Hypophthalmichthys molitrix* | 67457 | 64644 | 65088 | 111866 | 126225 | 118800 | 89564 | 19247 | 20492 | 69370 | 77294 | 72384 |  |
| *Misgurnus anguillicaudatus* | 401981 | 435449 | 399221 | 282977 | 239365 | 288626 | 389689 | 408217 | 420077 | 317138 | 316046 | 317270 |  |
| *Pseudorasbora parva* | 9631 | 11043 | 13779 | 26121 | 30265 | 25405 | 6852 | 8742 | 11838 | 3660 | 4465 | 4867 |  |
|  |  |  |  |  |  |  |  |  |  |  | total reads： | 8,903,288 |  |

| Table S3. Averaged reads for each species of each triplicate sample detected with four primer pairs in four tanks. | | | | | | | | |
| --- | --- | --- | --- | --- | --- | --- | --- | --- |
| **Species** | **Tank 1** | | | | **Tank 2** | | | |
|  | **AcMDB07** | **MiFish-U** | **Ve16S1** | **Ve16S3** | **AcMDB07** | **MiFish-U** | **Ve16S1** | **Ve16S3** |
| *Carassius auratus* | 1866 | 127284 | 52666 | 103456 | 7148 | 136530 | 69977 | 141483 |
| *Cyprinus carpio* | 2310 | 117083 | 82724 | 151317 | 3640 | 54675 | 51330 | 85589 |
| *Gambusia affinis* | 27583 | 37974 | 11705 | 10865 | 133701 | 175069 | 80339 | 83091 |
| *Hypophthalmichthys molitrix* | 143895 | 88210 | 61832 | 65730 | 257558 | 138499 | 119273 | 118964 |
| *Misgurnus anguillicaudatus* | 493084 | 377777 | 333290 | 412217 | 267595 | 239228 | 208305 | 270323 |
| *Pseudorasbora parva* | 12433 | 10705 | 5316 | 11484 | 24993 | 19026 | 13709 | 27264 |
|  |  |  |  |  |  |  |  |  |
| **Species** | **Tank 3** | | | | **Tank 4** | | | |
|  | **AcMDB07** | **MiFish-U** | **Ve16S1** | **Ve16S3** | **AcMDB07** | **MiFish-U** | **Ve16S1** | **Ve16S3** |
| *Carassius auratus* | 1758 | 114158 | 46727 | 90576 | 6930 | 263568 | 126478 | 256230 |
| *Cyprinus carpio* | 3040 | 145061 | 102920 | 178489 | 1382 | 40308 | 46152 | 63129 |
| *Gambusia affinis* | 43871 | 55153 | 18329 | 16257 | 61484 | 69180 | 26991 | 28897 |
| *Hypophthalmichthys molitrix* | 103802 | 65903 | 48438 | 43101 | 191198 | 94143 | 84765 | 73016 |
| *Misgurnus anguillicaudatus* | 539196 | 370919 | 335753 | 405994 | 391759 | 282627 | 275248 | 316818 |
| *Pseudorasbora parva* | 9916 | 8303 | 4244 | 9144 | 4776 | 2693 | 3610 | 4331 |
